# Biomonitoring 3.0: Beyond Taxa Lists to Evidence‐Tiered Monitoring of Ecosystem Dynamics and Interactions

**DOI:** 10.1002/ece3.74311

**Published:** 2026-09-06

**Authors:** Leandro Lofeu, Ehsan Pashay Ahi

**Affiliations:** ^1^ Department of Biology University of Sao Paulo Sao Paulo Brazil; ^2^ Organismal and Evolutionary Biology Research Programme, Faculty of Biological and Environmental Sciences University of Helsinki Helsinki Finland; ^3^ Institute of Biomedicine Faculty of Medicine, University of Turku Turku Finland

**Keywords:** biomonitoring 3.0, cross‐kingdom interactions, ecological networks, environmental RNA, environmental transcriptomics, interaction‐ready monitoring, time‐resolved ecosystem dynamics

## Abstract

High‐throughput sequencing has transformed biomonitoring by enabling scalable molecular inventories, but most programs still emphasize taxa lists. In this Perspective, Biomonitoring 3.0 is proposed as a framework that builds on molecular inventories to monitor ecological dynamics, interactions, and biological responses through time. Environmental RNA is presented as a complementary source of information because, in some settings, it can provide more temporally local evidence of recent biological activity and responses than DNA alone. An inference ladder is introduced to grade interaction evidence from co‐detection and statistical associations to interaction‐explicit links, coupled signal–response dynamics and, where feasible, ecosystem‐level consequences. Field designs are outlined that use repeated time‐series sampling and paired sampling of potential signal sources and recipients to improve temporal interpretation and strengthen attribution. Minimum reporting elements are also recommended to support transparent comparison, validation, and cross‐study synthesis. The “3.0” designation therefore refers to a change in the primary monitoring objective: from documenting community membership alone to evaluating ecological dynamics and feedbacks relevant to ecosystem condition and management. We conclude with a practical agenda for developing environmental nucleic‐acid measurements into decision‐relevant indicators of interactions and change.

## Graphical abstract

Biomonitoring has scaled rapidly with high‐throughput sequencing, but most programs still culminate in taxa lists that are weakly linked to the interaction dynamics driving ecosystem change. Here we propose Biomonitoring 3.0 as interaction‐ready, time‐resolved monitoring that layers eDNA inventories with time‐structured sampling, interaction‐explicit evidence channels, and, where informative, environmental RNA to improve temporal localization and capture organismal/community responses. We introduce an interaction evidence ladder (Tier 0–5) and outline field‐design and reporting priorities that make interaction claims auditable, comparable, and decision‐relevant.

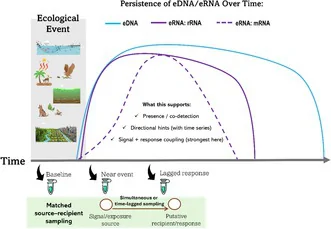

## 1 Introduction: Why Biomonitoring 3.0 Now

Ecosystem condition has traditionally been inferred from standardized field sampling of indicator assemblages and the scoring of biotic indices based on morphology‐driven identifications (Haase et al. 2010; Pawlowski et al. 2018; Simaika et al. 2024). Persistent limitations have been documented in cross‐program harmonization, quality assurance, and long‐term comparability, and substantial error has been traced to sample processing and taxonomic identification steps (Haase et al. 2010; Simaika et al. 2024). As a result, routine bioassessment has often been constrained by limited taxonomic capacity, delayed reporting, and reduced sensitivity to rapid or subtle ecological change (Haase et al. 2010; Pawlowski et al. 2018; Simaika et al. 2024).

A major inflection point has been created by high‐throughput sequencing and DNA‐based identification, through which the “taxonomic bottleneck” has been targeted at scale (Baird and Hajibabaei 2012; Taberlet et al. 2012). Biomonitoring 2.0 was articulated as a paradigm in which DNA‐based identification is coupled to next‐generation sequencing to increase the information extracted from routine biomonitoring samples while reducing reliance on scarce taxonomic expertise (Baird and Hajibabaei 2012). In parallel, DNA metabarcoding has been advanced as a general approach for multi‐taxon identification from bulk and environmental samples, while being explicitly framed as dependent on marker choice, reference databases, and workflow standardization (Pawlowski et al. 2020, 2021; Taberlet et al. 2012). In the decade since that framing, molecular biomonitoring has been expanded from pilot studies to coordinated applications, while methodological heterogeneity, interpretation limits, and transparency of reporting have remained recurring constraints on uptake and synthesis (Blackman et al. 2024; Pawlowski et al. 2020, 2021).

Despite this maturation, routine molecular monitoring applications have frequently remained dominated by taxa‐by‐sample inventories and status‐class assignment derived primarily from community membership (Blackman et al. 2024; Pawlowski et al. 2018, 2020, 2021). This emphasis reflects prevailing implementation rather than an intrinsic informational limit of eDNA: eDNA can support c and population‐genetic inference, while molecular‐state and emerging methylation‐based information may add temporal, demographic, physiological, and life‐history context (Jo et al. 2023; Liu et al. 2026; Ramón‐Laca et al. 2026). Ecological attribution has also been complicated by the breadth of “environmental DNA” as a term, which has been shown to encompass multiple biological sources, transport pathways, and mixtures of intracellular and extracellular DNA across the tree of life (Blackman et al. 2024; Pawlowski et al. 2020). Legacy signal and transport‐mediated detection have therefore been treated as practical challenges for site‐specific inference, particularly when short time windows or near‐real‐time interpretation are required (Blackman et al. 2024; Pawlowski et al. 2020).

A further limitation has been imposed by the weak mechanistic link between inventories and ecosystem function: change has often been mediated through altered species interactions, and early disturbance has frequently been expressed as shifts in interaction structure or interaction loss before consistent species turnover is observed (Delmas et al. 2019; Tylianakis et al. 2008; Valiente‐Banuet et al. 2015). Ecological network analysis has been established as a framework for formalizing interaction structure and comparing community organization across contexts (Delmas et al. 2019). However, routine translation from molecular inventories to interaction inference has remained non‐trivial, and “next‐generation biomonitoring” concepts based on network reconstruction have been accompanied by explicit cautions regarding spurious links, sensitivity to sampling design, and interpretability of inferred edges (Barroso‐Bergadà et al. 2021).

Environmental RNA (eRNA) has increasingly been proposed as a complementary layer through which monitoring can be shifted toward shorter time scales and closer coupling to biological state (Yates et al. 2021). Faster decay of eRNA relative to environmental DNA has been quantified under varying pH and temperature conditions, while also showing that persistence is environmentally dependent (Jo et al. 2023; Kagzi et al. 2022). In addition, joint RNA/DNA dynamics have been shown to change predictably during degradation in controlled experiments, and eRNA:eDNA ratios have been proposed as one route to estimate the age or recentness of environmental nucleic acids under defined conditions (Marshall et al. 2021). Beyond recency, environmental transcriptomics has been demonstrated to recover gene expression responses of macroorganisms and communities from extra‐organismal RNA, including in controlled exposures and mesocosm settings designed to emulate environmental change (He et al. 2025; Hechler et al. 2023; Hiki et al. 2023; Kagzi et al. 2022).

On this basis, Biomonitoring 3.0 is proposed here as a decision‐led, molecule‐agnostic framework that retains molecular inventories as a foundation while directing the monitoring question, design, and reported output toward ecosystem dynamics, interactions, or biological responses (Abdala‐Roberts et al. 2025; Barroso‐Bergadà et al. 2021; Delmas et al. 2019; Yates et al. 2021). A program meets this definition when it (i) specifies a decision‐relevant hypothesis about dynamics, interactions, or responses; (ii) uses temporal, interaction‐explicit, response, or process evidence appropriate to that hypothesis; and (iii) reports the achieved evidence tier and its uncertainty. eRNA is a central enabling layer within this framework, particularly for time‐local activity and response readouts, but its use alone does not define Biomonitoring 3.0. A shorter detection window alone is therefore a methodological refinement; it contributes to Biomonitoring 3.0 only when it is linked to a dynamics‐focused question and an explicit inference and decision pathway. The “3.0” designation is intended to mirror the earlier 2.0 framing while marking a distinct monitoring object—ecosystem dynamics and interaction state—rather than a further scaling of taxon enumeration alone (Baird and Hajibabaei 2012; Barroso‐Bergadà et al. 2021; He et al. 2025; Hechler et al. 2023; Hiki et al. 2023). A practical prerequisite for this shift is indicated by the growing emphasis on minimum‐information reporting for environmental metabarcoding, through which cross‐study synthesis and benchmarking are supported and interaction claims can be evaluated on reproducible evidence tiers rather than on terminology (Klymus et al. 2024).

## 2 From Taxa Lists to Interaction‐Ready Inference: What Changes in 3.0

Three distinct monitoring “targets” have typically been pursued with molecular data: community membership (taxa lists), community function (genes/transcripts and associated pathways), and community structure as an interaction system (who is linked to whom, and how those links shift) (Cordier et al. 2021). A practical classification of implementation strategies has already been formalized in environmental genomics, where taxonomy‐based inventories, de novo indicator discovery, structural community metrics (including inferred ecological networks), and functional community metrics (metagenomics or metatranscriptomics) have been treated as separable routes to ecological diagnosis (Cordier et al. 2021). Under this framing, the conceptual shift proposed for biomonitoring 3.0 has been expressed as a change in the primary object of inference—from static membership toward interaction state and short‐timescale dynamics—while being kept compatible with existing inventory‐based workflows (Bohan et al. 2017; Cordier et al. 2021). The boundary is therefore defined by the monitoring objective, evidence design, and reported claim rather than by molecule choice: the use of eRNA alone does not constitute Biomonitoring 3.0, and eDNA‐based or other molecular programs can meet the framework when they test and grade hypotheses about dynamics, interactions, responses, or process change.

The motivation for privileging interactions has been supported by long‐standing ecological reasoning: ecosystem change is frequently mediated through altered trophic, competitive, mutualistic, or parasitic relationships rather than through immediate extirpations, and management leverage is often exerted through the rewiring of those relationships (Abdala‐Roberts et al. 2025; Delmas et al. 2019). Ecological network analysis has therefore been promoted as a mechanistic scaffold for describing community organization and for quantifying changes in ecosystem structure in ways that cannot be recovered from unlinked taxa lists alone (Delmas et al. 2019). A monitoring vision has even been articulated in which nucleic acids sampled from environments are used—together with automated analytics—to reconstruct ecological interaction networks at broad spatial and temporal scales (Bohan et al. 2017). Within “next‐generation biomonitoring” agendas, network reconstruction from sequencing data has been positioned as a route to earlier and more generalizable detection of ecosystem change than would be achieved by indicator taxa alone (Makiola et al. 2020).

At the same time, interaction inference from routine molecular inventories has been shown to be scientifically fragile unless the limits of observational data are treated explicitly. Co‐occurrence patterns have been demonstrated to be an unreliable proxy for ecological interaction when confounding processes (shared habitat preference, sampling artifacts, indirect effects, compositionality, and detectability) are present (Blanchet et al. 2020). For metabarcoding data specifically, taxon‐specific PCR amplification efficiencies can distort relative sequence‐read proportions, requiring additional caution when compositional read data are used to characterize community structure or support downstream ecological inference (Gold et al. 2023; Kelly et al. 2019; Shelton et al. 2023). In a targeted examination of network inference for biomonitoring, strengths have been acknowledged (scalability and sensitivity), but spurious edges, instability to sampling design, and ambiguous ecological meaning have been emphasized as central pitfalls (Barroso‐Bergadà et al. 2021). Related caution has been raised for metabarcoding generally, where robust experimental design has been argued to be essential for sound ecological inference, particularly when complex conclusions (including network claims) are drawn from amplicon data (Zinger et al. 2019). Even when more structured statistical approaches are used, limits have been quantified: joint species distribution models have been shown to detect some interaction types under simulation but to fail for predator–prey interactions under conditions where residual correlation does not map cleanly to interaction coefficients (Zurell et al. 2018). Interaction‐ready inference has therefore been best viewed as a design goal rather than as a default output of metabarcoding. In this article, the term has been used to denote monitoring programs in which sampling, metadata, and analysis are configured so that interaction hypotheses can be tested and graded rather than merely suggested by correlation. This goal has been approached in the literature through at least three complementary strategies.

First, time‐series and replicated sampling have been used to constrain interaction inference by requiring temporal coherence and repeated edge recovery. In a high‐impact example, eDNA time‐series in a marine system have been used to infer seasonal shifts and putative interactions (including expected trophic linkages) across domains of life, with network patterns interpreted alongside environmental variables and community turnover (Djurhuus et al. 2020). Such work has illustrated both the promise and the vulnerability of network interpretation: dynamic structure has been detected, but mechanistic directionality has remained dependent on external knowledge and validation (Djurhuus et al. 2020). Greater temporal resolution can strengthen evidence for temporal ordering, but it does not by itself convert observational association into evidence of causality. Second, metaweb and trait‐ or literature‐informed constraints have been used to translate inventories into plausible interaction structure. In marine settings, rapid reconstruction of food webs from eDNA‐derived inventories has been operationalized by combining molecular detections, which can increasingly include quantitative multispecies indices (Guri et al. 2025), with curated trophic information, thereby turning taxa lists into potential networks that can be summarized by network indicators (D'Alessandro and Mariani 2021; Marie‐Anne Le Guen et al. 2025). Recent work has explicitly formalized this approach for ecosystem‐health assessment by deriving food web indicators (e.g., connectivity and redundancy) from food webs reconstructed using eDNA (Marie‐Anne Le Guen et al. 2025). In a closely related direction, co‐occurrence networks derived from eDNA have been evaluated against food web expectations and metaweb information in a coastal marine system, with support reported for recovering some expected links while underscoring the need for careful validation and interpretation (Boyse et al. 2025).

Third, interaction evidence has been strengthened by sampling designs that directly encode interactions, rather than inferring them from shared presence. Diet metabarcoding and related molecular approaches have been used to parameterize trophic links and even energetic food web models, with DNA‐based feeding selectivity being integrated into energy‐flux estimation (Novotny et al. 2023; Pompanon et al. 2012). For mutualistic networks, pollen DNA metabarcoding has been used to reconstruct plant–pollinator interaction networks and has been shown to reveal cryptic diversity and high spatial turnover in interactions, with “rewiring” quantified across habitats (Encinas‐Viso et al. 2023). These interaction‐explicit channels have been particularly useful because link meaning is less ambiguous than in co‐occurrence graphs, even though quantitative biases and reference‐database dependence have still required explicit handling (Deagle et al. 2019; Novotny et al. 2023; Zinger et al. 2019).

Taken together, these strands have suggested that the practical distinction between biomonitoring 2.0 and 3.0 is not an incremental increase in the number of taxa detected, but a change in what is treated as the core monitoring output. In biomonitoring 3.0, inventories are not discarded; instead, they are used as scaffolding onto which interaction‐explicit data streams, network constraints, and replicated temporal designs are layered so that ecosystem change can be tracked as a shift in links and feedbacks rather than only as a shift in membership (Barroso‐Bergadà et al. 2021; Bohan et al. 2017; Cordier et al. 2021; Makiola et al. 2020; Zinger et al. 2019). The corresponding decision advance is not simply earlier detection; it is the ability to identify the timing and response context of ecological change, distinguish association‐level from interaction‐explicit evidence, and match management action to the strength of the supported claim. The role of eRNA in making these outputs more time‐resolved (and, in some contexts, more closely tied to biological activity) is introduced in the next section to avoid conflating interaction inference with molecule choice (Cordier et al. 2021).

## 3 eRNA as a Time‐Resolved Layer: From Presence to Activity and Response

eRNA has been proposed as an extension of environmental nucleic‐acid monitoring in which signal recency and biological state are emphasized rather than treated as secondary interpretation issues (Yates et al. 2021). Importantly, eRNA does not represent a single, uniform signal class: different RNA types and molecular states can vary substantially in their abundance, persistence, and biological interpretation (Ahi and Schenekar 2025; Yates et al. 2021). A conceptual distinction has been drawn between the broad detectability of environmental DNA (eDNA) and the potentially shorter persistence and closer state‐coupling of some RNA fractions, with the latter being framed as a route to more temporally local inference when rapid ecological change is of concern (Yates et al. 2021). Empirical support for “time‐resolved” inference has been strengthened by comparative decay experiments in which faster loss of eRNA than eDNA has been quantified under controlled conditions (Kagzi et al. 2022). Across a broad pH gradient, detectability windows were reported to be far shorter for eRNA than for eDNA when modeled as exponential decay, with eRNA detectability predicted to extend up to ~57 h (in that experiment) rather than weeks to months, while eDNA detectability was predicted to extend up to ~143 days under the conditions tested (Kagzi et al. 2022). A controlled marine decay experiment using seawater from an open‐net dolphin enclosure similarly showed strong molecule‐dependent persistence, with messenger eRNA disappearing rapidly while ribosomal eRNA persisted longer and decayed only slightly faster than its corresponding eDNA marker (Brandão‐Dias et al. 2025). Field‐relevant mesocosm work has further shown that the rate contrast between eRNA and eDNA can persist under complex community settings and across multiple genetic markers, while also demonstrating that decay kinetics are not uniform across transcript types (Morgado‐Gamero et al. 2025). In a dPCR‐based comparison across connected and isolated 1000‐L mesocosms, faster degradation of eRNA was reported across markers and habitats, and faster decay of messenger RNA (mRNA) targets than ribosomal RNA (rRNA) targets was documented, indicating that “time resolution” is partly governed by the choice of transcript class as well as by the environment (Morgado‐Gamero et al. 2025). In the same study, dilution through hydrologic connectivity was shown to reduce detectability while not eliminating it, implying that both decay and transport must be accounted for when time‐resolved interpretations are attempted at landscape scales (Morgado‐Gamero et al. 2025).

Beyond signal recency, some eRNA applications have explored whether particular RNA targets can preferentially reflect living or recently active components of the community signal (Yates et al. 2021). However, such interpretation depends on the RNA target, organism, environmental context, and analytical workflow. In river bioassessment and water‐quality contexts, higher positive predictivity (lower apparent false‐positive tendency) has been reported for eRNA relative to eDNA under several analytical configurations, even when sensitivity was reduced for some taxa (Miyata et al. 2022). A large field evaluation spanning nine organismal groups from bacteria to terrestrial vertebrates has likewise documented robust complementarity between eDNA and eRNA, with systematic dependence of relative detectability on organismal size and taxonomic group (Zhang et al. 2024). In that study, slightly higher apparent false‐positive rates were reported for eDNA than for eRNA, while higher false‐negative rates were reported for eRNA. Such terminology requires careful interpretation because a valid molecular detection outside the contemporaneous spatial or temporal occurrence of the target organism may produce an erroneous ecological inference without constituting an analytical false‐positive detection (Darling et al. 2021). Field sampling in freshwater lakes has also demonstrated that extra‐organismal eRNA from macroeukaryotes can be recovered and metabarcoded using approaches comparable to eDNA, with broadly similar characterization of fish‐community composition (Littlefair et al. 2022). Similar tradeoffs have been observed in other comparative field studies, where a more localized vertebrate signal has been attributed to eRNA in some contexts while stronger invertebrate recovery has been retained for eDNA depending on marker and target group (Macher et al. 2024). In marine and estuarine settings, community differences have been detected between eRNA‐ and eDNA‐templated metabarcoding, and eRNA has been discussed as particularly useful where legacy DNA is expected to inflate detections of organisms no longer present (Giroux et al. 2022).

Evidence for reduced “ghost” detection has also been developed in biosecurity and applied monitoring settings where dead biomass and extracellular DNA are expected to be abundant (Xue et al. 2024). In ballast‐water monitoring before and after disinfection, eRNA‐based 16S metabarcoding was reported to recover broadly similar high‐abundance community structure while producing fewer detections that were interpreted as legacy signal from dead cells, yielding a lower apparent false‐positive tendency in treated samples (Xue et al. 2024). In fish‐focused surveys, performance gains for eRNA over eDNA have been reported in several river applications when evaluated against traditional field surveys, and higher positive predictivity has been highlighted as a practical advantage for management‐facing interpretation (Miyata et al. 2021, 2025). At the same time, reduced sensitivity and higher false‐negative propensity have been repeatedly flagged as realistic constraints on eRNA deployments, particularly when organism densities are low, RNA preservation is imperfect, or sequencing depth is insufficient (Macher et al. 2024; Miyata et al. 2022; Zhang et al. 2024).

A second, and more distinctive, contribution of eRNA to biomonitoring 3.0 has been made through readouts of biological activity and transcriptional responses to environmental change that complement taxonomic DNA inventories (Ahi et al. 2025; Yates et al. 2021). Environmental transcriptomics has been used to recover macroorganism gene‐expression responses from extra‐organismal RNA without direct organism sampling, and concordant directionality between organismal RNA and extra‐organismal eRNA has been reported for subsets of heat‐stress‐responsive genes in controlled aquatic experiments (Hechler et al. 2023). In that work, thousands of target‐organism genes were detected in tank water, differential expression of heat‐stress‐relevant genes was recovered, and community‐wide shifts in functional profiles were detected, showing that response information can be carried in environmental matrices alongside taxonomic signals (Hechler et al. 2023). Chemical‐stressor sensitivity has been demonstrated in parallel: differential expression following sublethal pyrene exposure was detected from eRNA released by Japanese medaka, supporting a noninvasive path to hazard‐relevant response monitoring at the transcript level (Hiki et al. 2023). At the community scale, eRNA‐based metatranscriptomics has been used in outdoor mesocosms to track herbicide‐associated transcriptional change across diverse freshwater eukaryotes spanning multiple trophic levels, with differential expression and pathway enrichment being evaluated at the class level (He et al. 2025).

These demonstrations have also clarified why response‐oriented eRNA monitoring has not yet been routine. In the heat‐stress environmental transcriptomics study, only a small fraction of total eRNA reads mapped to the focal macroorganism genome, and the “competition” between extra‐organismal macroorganism RNA and abundant microbial RNA was explicitly treated as a key limitation for translation to natural systems (Hechler et al. 2023). Methodological routes for addressing this imbalance have been discussed within the primary literature and in application syntheses, including deeper sequencing, rRNA depletion, poly(A) enrichment for eukaryotic mRNA, larger‐pore filtration under selected conditions, and targeted assay development for diagnostic transcripts (Costea et al. 2026; Hechler et al. 2023). These approaches can improve recovery of target transcripts but do not guarantee sufficient signal from macro‐organisms in complex field samples, particularly when target RNA is scarce, and therefore do not remove the risk of false negatives. At present, untargeted environmental transcriptomics for macro‐organism response monitoring should therefore be regarded as an emerging rather than routine monitoring approach, with targeted or enrichment‐based strategies likely to be more practical when specific response questions and candidate transcripts are available (Duenser and Ahi 2026). This limitation should be distinguished from taxonomic and targeted eRNA applications that have already been demonstrated in natural systems (Miyata et al. 2021, 2025; Parsley and Goldberg 2024). For response‐oriented environmental transcriptomics, however, requirements for sequencing depth, enrichment, RNA preservation, and downstream analysis currently increase cost and technical complexity and constrain routine long‐term deployment. A conservation‐ and management‐facing synthesis of eRNA targets has formalized this logic by proposing criteria for gene target selection and by cataloging candidate genes intended to support demographic inference, stress detection, and other applied questions (Khorshid et al. 2026; Stevens and Parsley 2023). Population‐demographic information has also been extracted directly in some cases: life‐stage discrimination in amphibian populations has been demonstrated using field‐collected eRNA, indicating that activity and response monitoring can be extended beyond stress pathways to traits with direct management relevance (Lofeu and Ahi 2026; Parsley and Goldberg 2024).

A third reason for treating eRNA as a biomonitoring 3.0 layer has been provided by its molecular diversity. More broadly, environmental nucleic acids can provide multiple information layers beyond taxonomic detection, including abundance, population, physiological, and life‐history information, with eDNA methylation emerging as a route to population‐level trait inference (Liu et al. 2026; Ramón‐Laca et al. 2026). While most current eRNA biomonitoring has been centered on rRNA templates for metabarcoding and mRNA templates for transcriptomics, a broader landscape of RNA types and RNA‐associated processes has been argued to remain underused in environmental applications (Ahi and Schenekar 2025; Pashay Ahi et al. 2025). A recent Molecular Ecology roadmap has emphasized that regulatory and structural RNAs beyond mRNA can encode information about stress responses, development, and adaptation, and that methodological and bioinformatic development will be required if these signals are to be harnessed reproducibly from environmental matrices (Ahi and Schenekar 2025). This argument has been aligned with biomonitoring 3.0 because interaction‐ready, time‐resolved inference is expected to benefit from signals that change quickly and map more directly onto organismal state than presence/absence alone (Yates et al. 2021).

**FIGURE 1:**
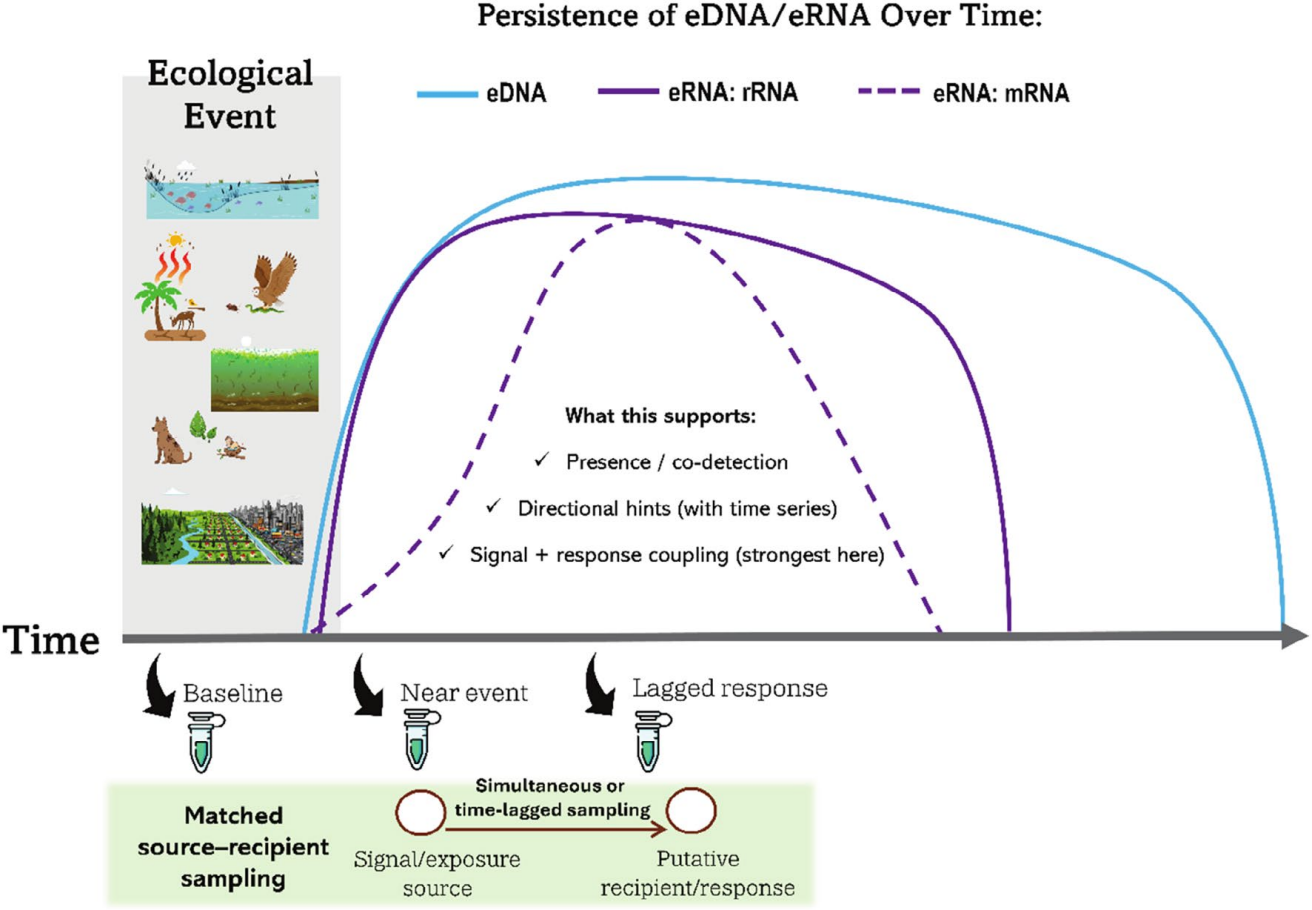
Temporal persistence and inferential value of environmental DNA (eDNA) and environmental RNA (eRNA). After an ecological event (left), eDNA (solid blue curve, inventory signal) rises and remains detectable for longer, providing a broad and temporally extended detection window. eRNA rRNA (solid purple curve, recent community signal) shows a similar pattern but declines sooner, making it more temporally localized. eRNA mRNA (dashed purple curve, recipient response) peaks later, representing a short‐lived, delayed response that may provide a stronger link to contemporaneous biological activity. Sampling across baseline, near‐event, and lagged‐response phases enables inference of presence, co‐detection, and directional change through time. The strongest signal–response coupling is expected for short‐lived RNA fractions. In this schematic, matched source–recipient sampling refers to corresponding samples collected from a putative signal or exposure source and an ecologically connected recipient, either simultaneously or after a predefined time lag. This design allows evaluation of whether a source‐associated signal is followed by a biological response in the recipient, potentially detected through eRNA. The source–recipient pairing shown here is distinct from paired eDNA and eRNA measurements obtained from the same environmental sample.

Taken together, the role of eRNA in biomonitoring 3.0 has not been justified by a blanket claim that RNA is better than DNA, but by a set of empirically supported tradeoffs that are advantageous when rapid dynamics and state inference are required. Controlled decay studies have shown faster eRNA than eDNA decay under specified conditions, while persistence can also differ among transcript classes and environmental settings (Brandão‐Dias et al. 2025; Kagzi et al. 2022; Morgado‐Gamero et al. 2025). These differences can support more time‐local interpretation, but only when persistence is calibrated for the target molecule and sampling context. Comparative field studies have supported complementarity between eDNA and eRNA and identified context‐dependent differences in detection performance (Macher et al. 2024; Miyata et al. 2022; Xue et al. 2024; Zhang et al. 2024). Most importantly for the shift from presence to process, extra‐organismal transcriptomics has shown that response information can be recovered noninvasively from environmental media, albeit with clear requirements for sequencing depth, enrichment, and careful assay design (Hechler et al. 2023). How these properties can be converted into interaction‐ready monitoring, through coupling signal sources to recipient responses and through explicitly time‐lagged sampling, has been developed in the subsequent section as a design problem rather than as an intrinsic guarantee of any single molecule type. This timing logic; event, persistence, and lagged response, is summarized schematically (Figure 1).

## 4 What Counts as an Interaction Signal: An Evidence Ladder for Claims

The term interaction has often been applied loosely in molecular biomonitoring, where correlations among detections are sometimes interpreted as ecological links without explicit separation of association, causation, and mechanism (Bohan et al. 2017; Cordier et al. 2021; Delmas et al. 2019). A persistent risk has therefore been created for biomonitoring 3.0: interaction‐ready outputs can be demanded conceptually, yet the evidentiary basis behind an “interaction signal” can remain ambiguous unless explicit inference tiers are adopted (Abdala‐Roberts et al. 2025; Barroso‐Bergadà et al. 2021; Bohan et al. 2017; Delmas et al. 2019). In ecology more broadly, caution has been urged because predictive or associative models have frequently been misread as causal explanations, particularly when monitoring data are opportunistic, sparse, or confounded by shared drivers (Schrodt et al. 2025).

A pragmatic solution has been proposed here in the form of an interaction evidence ladder, through which claims can be graded by the kind of information that is required and by the most likely failure modes at each tier (Bohan et al. 2017; Cordier et al. 2021; Delmas et al. 2019; Schrodt et al. 2025) (the ladder and its practical requirements are summarized in Table 1). Related arguments have been made repeatedly in network ecology and microbial network inference: network structure can be informative, but link meaning is method‐dependent, and confidence should be scaled to data structure and validation strength rather than to network aesthetics (Barroso‐Bergadà et al. 2021; Cordier et al. 2021; Delmas et al. 2019; Schrodt et al. 2025; Zinger et al. 2019). By placing interaction claims on an explicit ladder, biomonitoring 3.0 can be kept safe in two ways: (i) weak evidence can be reported without being oversold, and (ii) stronger evidence can be pursued intentionally through sampling and validation design (Barroso‐Bergadà et al. 2021; Schrodt et al. 2025; Zinger et al. 2019).

**TABLE 1:** Interaction evidence ladder for Biomonitoring 3.0. Summary of interaction‐claim tiers (0–5) and what each tier can legitimately support, paired with minimum design/data requirements and common failure modes. The table is intended as a reporting and planning aid: Programs should specify an intended tier before sampling, assign an achieved tier after data‐quality and assumption checks, and select sampling designs that can realistically meet the tier's assumptions. If those assumptions are not met, the result should be reported at the highest defensible tier supported by the realized data.

Tier	What you can claim	Minimum data/design requirements	Typical pitfalls/failure modes	Example Biomonitoring 3.0 outputs	Key refs (non‐exhaustive)
0	Co‐detection/association in space–time	Cross‐sectional samples + controls	Habitat filtering, transport, detectability artifacts	Taxa A and B co‐occur seasonally	Blanchet et al. (2020), Zinger et al. (2019)
1	Conditional association network (not mechanism)	Replication + confounder control; uncertainty/stability checks	Compositionality, sparsity, method dependence	Network metrics w/bootstrap stability	Barroso‐Bergadà et al. (2021), Weiss et al. (2016), Kurtz et al. (2015)
2	Directional association suggested by time ordering	Time series with adequate frequency, covariates, replication	Unmeasured confounders; aliasing; nonstationarity	Candidate driver → response edges	Runge et al. (2019), Schrodt et al. (2025), Sugihara et al. (2012)
3	Interaction‐explicit links (edge meaning is clear)	Diet/pollen/gut/parasite‐on‐host designs	Quantitative bias; contamination; ref. DB gaps	Trophic or mutualistic networks with explicit edges	Cordier et al. (2021), Pompanon et al. (2012), Deagle et al. (2019), Encinas‐Viso et al. (2023)
4	Coupled signal–response consistent with mechanism (not causal proof)	Paired source/recipient + time lag + response readout (often eRNA)	Low target mapping fraction; batch effects; alternative drivers	Stress signature in recipient aligned to exposure	Hechler et al. (2023), Hiki et al. (2023), He et al. 2025, Oña et al. (2025)
5	Ecological consequence (process‐level change)	Process metrics + validation/perturbation + robust linkage	Process multi‐causality; scale mismatch	Link shifts associated with productivity/stability proxies	Delmas et al. (2019), Thébault and Fontaine (2010), Allesina and Tang (2012), Abdala‐Roberts et al. (2025)

*Note:* Operational note: The tiers are not sequential. When sparse or discontinuous time series cannot support temporal ordering, claims are limited to Tier 0–1 unless an independent interaction‐explicit evidence channel supports Tier 3. Tier 4–5 require aligned response or process evidence.

### 4.1 Tier 0: Co‐Detection and Co‐Occurrence (Signals of Shared Space or Time)

At the lowest tier, an interaction signal has been limited to co‐detection or spatial association among taxa in the same samples or sites (Blanchet et al. 2020). Co‐occurrence has long been recognized as information‐rich for community assembly questions, but it has been argued explicitly that co‐occurrence should not be treated as evidence of interaction because association can be generated by shared habitat preference, dispersal history, sampling artifacts, detection limits, or indirect effects (Blanchet et al. 2020). When co‐occurrence has been used in monitoring, interpretation has therefore been advised to remain at the level of pattern description unless stronger constraints are added (Blanchet et al. 2020).

### 4.2 Tier 1: Association Networks With Confounder Control and Uncertainty Reporting

At the next tier, association networks have been inferred with explicit attention to confounding and statistical artifacts, and uncertainty has been treated as a required output rather than as an afterthought (Barroso‐Bergadà et al. 2021; Blanchet et al. 2020; Faust and Raes 2012; Zinger et al. 2019). Inferred microbial networks have been reviewed as useful summaries of community structure, yet high sensitivity has been reported to compositionality, sparsity, and pipeline choice, with spurious edges remaining a central concern even under careful modeling (Barroso‐Bergadà et al. 2021; Kurtz et al. 2015; Weiss et al. 2016; Zinger et al. 2019). For biomonitoring, it has been recommended that association networks be accompanied by stability checks (subsampling, alternative inference methods, and sensitivity analyses) and by a clear statement that the edges denote conditional associations rather than mechanistic interactions (Barroso‐Bergadà et al. 2021; Blanchet et al. 2020; Zinger et al. 2019).

### 4.3 Tier 2: Directional Association Suggested by Time Series and Causal Inference Tools

Directional evidence has been treated as a distinct tier because the step from associated to influential requires information on temporal ordering (or controlled perturbation) (Runge et al. 2019; Schrodt et al. 2025). In practice, directionality has been inferred from time series using causal discovery or dynamical‐systems methods, yet strong assumptions have been required about stationarity, sampling frequency, unmeasured confounders, and the adequacy of the observed variables (Runge et al. 2019; Schrodt et al. 2025; Sugihara et al. 2012). Accordingly, Tier 2 supports candidate directional relationships rather than causal interaction claims unless the assumptions required for causal attribution are independently justified. In Earth‐system applications, causal inference frameworks for time series have been synthesized and benchmarked, with explicit guidance provided on when causal graphs are likely to be unreliable (Runge et al. 2019). For ecological monitoring programs, recent practitioner‐oriented guidance has been published to separate change detection from causal attribution, and to align inference methods to data structure and the scale at which drivers and biodiversity responses are sampled (Schrodt et al. 2025). In biomonitoring 3.0, Tier 2 has therefore been treated as achievable only when sampling frequency, replication, and covariate coverage are sufficient to support causal assumptions, rather than being treated as a default upgrade over Tier 1 (Runge et al. 2019; Schrodt et al. 2025).

### 4.4 Tier 3: Interaction‐Explicit Links From Molecular Evidence Channels

Stronger interaction evidence has been obtained when links are measured directly rather than inferred indirectly from co‐occurrence (Cordier et al. 2021; Delmas et al. 2019). Diet metabarcoding, gut‐content sequencing, pollen DNA metabarcoding, parasite DNA detection on hosts, and similar designs have been used to encode interactions within the sampling itself, thereby reducing ambiguity about what an edge represents (Cordier et al. 2021; Delmas et al. 2019). In the biomonitoring context, next‐generation global biomonitoring concepts have explicitly highlighted the value of such interaction‐explicit evidence streams for network reconstruction at scale (Bohan et al. 2017). At this tier, the principal uncertainties have been shifted from “is an edge real?” toward “is the edge quantitative and comparable?”, because amplification bias, digestion bias, contamination, and reference‐library incompleteness can still distort link strength and apparent specificity (Blanchet et al. 2020; Cordier et al. 2021).

### 4.5 Tier 4: Coupled Signal–Response Evidence Consistent With Mechanism

A further evidentiary jump has been made when a putative signal is detected together with a recipient response that is consistent with a plausible mechanism, especially when time‐lag structure is observed (Runge et al. 2019; Schrodt et al. 2025). In microbial ecology, it has been emphasized that no single method can capture the full complexity of interaction networks, and that combinations of computational inference, mechanistic modeling, and experimental validation are often required (Oña et al. 2025). This logic has been adopted here for biomonitoring 3.0 by treating signal–response coupling as the defining feature of “interaction‐ready” monitoring: a candidate interaction has been supported more strongly when (i) exposure plausibility is established (co‐location in time/space and feasible transport), and (ii) a response signature is observed in the putative recipient that is temporally aligned and biologically interpretable, and alternative drivers are ruled out where feasible (Oña et al. 2025; Runge et al. 2019; Schrodt et al. 2025). eRNA has been positioned as particularly useful for Tier 4 because transcript profiles can function as response readouts, but Tier 4 has been kept molecule‐agnostic in principle to avoid conflating mechanistic inference with any single assay class (Schrodt et al. 2025). Even at this tier, signal–response coupling should be interpreted as evidence consistent with a mechanism rather than definitive causal proof unless supported by experimental perturbation or other independent validation.

### 4.6 Tier 5: Ecological Consequence Demonstrated at the Process Level

At the highest tier, interaction claims have been strengthened by demonstration that an inferred link (or a set of links) produces a measurable ecological consequence, such as altered productivity, stability, nutrient cycling, disease dynamics, or resilience metrics (Abdala‐Roberts et al. 2025; Delmas et al. 2019). Network topology has been tied theoretically and, in some systems, empirically to stability and function in microbial and broader ecological systems, and the need to connect inferred networks to process measurements has been emphasized as a key challenge for turning networks into decision tools (Allesina and Tang 2012; Coyte et al. 2015; Delmas et al. 2019; Oña et al. 2025; Thébault and Fontaine 2010). In applied contexts, management approaches have been argued to benefit from explicit interaction‐network thinking because leverage is often exerted through link modification rather than through species removal alone (Abdala‐Roberts et al. 2025). Tier 5 has therefore been framed as the long‐term target for biomonitoring 3.0 validation programs, while being acknowledged as infeasible to demonstrate routinely for every monitored system (Abdala‐Roberts et al. 2025; Schrodt et al. 2025).

### 4.7 Implications for Reporting and Interpretation in Biomonitoring 3.0

**FIGURE 2:**
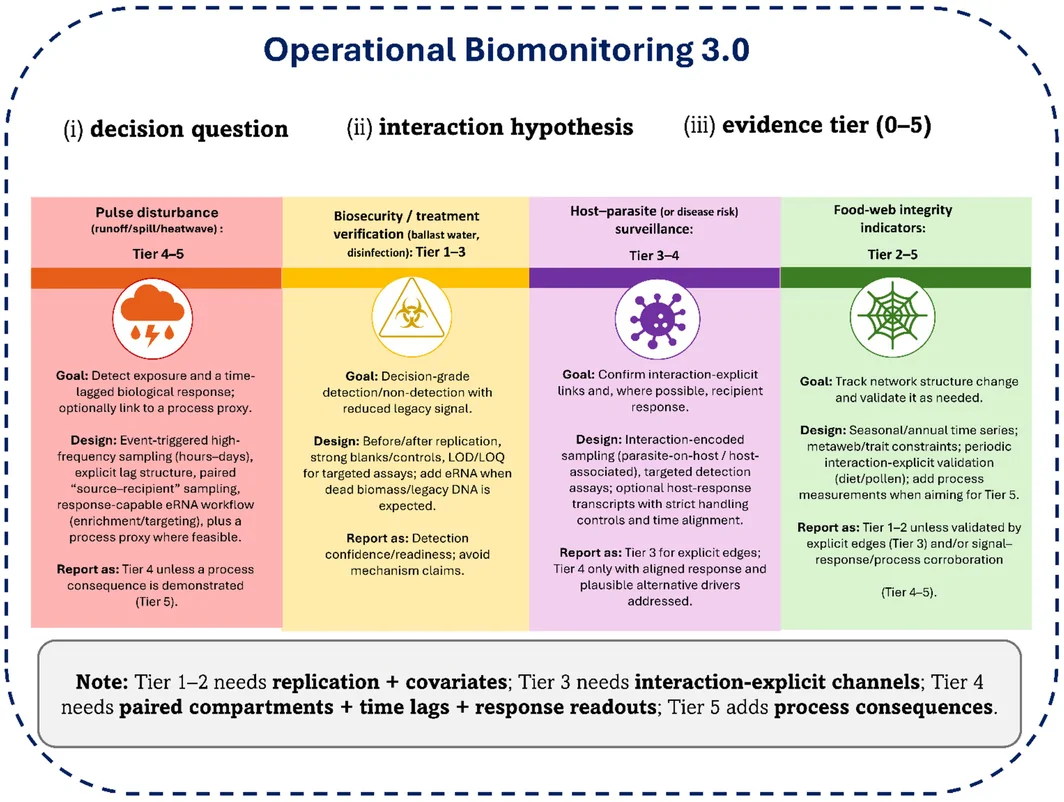
Operational framework for Biomonitoring 3.0 based on decision‐driven design and evidence tiers. The figure outlines how biomonitoring programs become operational by first defining (i) the decision question, (ii) the interaction hypothesis, and (iii) the intended evidence tier (0–5). Four representative use cases are shown: Pulse disturbance (Tier 4–5), biosecurity/treatment verification (Tier 1–3), host–parasite or disease‐risk surveillance (Tier 3–4), and food‐web integrity indicators (Tier 2–5). For each, the figure specifies the primary goal, recommended sampling and assay design elements, and how results should be reported in terms of evidentiary claims. Lower tiers emphasize replication and covariates, whereas higher tiers require interaction‐explicit channels, paired compartments, time lags, response readouts, and, at Tier 5, demonstrated process consequences.

Two operational recommendations have been derived from the ladder. First, interaction results should be reported with an explicit tier label (Tier 0–5), so that a monitoring outcome can be interpreted without assuming more than has been supported (Barroso‐Bergadà et al. 2021; Blanchet et al. 2020; Schrodt et al. 2025; Zinger et al. 2019). Second, sampling and analysis should be chosen with an intended tier in mind: Tier 1 has been attainable from well‐designed cross‐sectional sampling with strong controls, whereas Tier 2 requires adequate temporal replication and covariate coverage, Tier 3 requires interaction‐explicit evidence, and Tier 4 requires temporally aligned, mechanism‐linked measurements (Barroso‐Bergadà et al. 2021; Bohan et al. 2017; Runge et al. 2019; Schrodt et al. 2025; Zinger et al. 2019). The tiers are evidence categories rather than mandatory sequential steps or a simple gradient of cost and complexity: a focused interaction‐explicit design may support Tier 3 without first achieving Tier 2, while many routine programs may appropriately remain at Tiers 0–1. Programs should distinguish the intended tier specified before sampling from the achieved tier supported after data‐quality and assumption checks. Sparse, discontinuous, or poorly replicated data set an upper limit on the defensible tier. When the minimum requirements for the intended tier are not met, results should be reported at the highest tier supported by the realized data rather than being elevated solely through interpolation or increased model complexity. Higher‐tier measurements may also be deployed selectively or periodically for calibration and validation rather than continuously. By adopting this ladder as a shared language, biomonitoring 3.0 can be advanced as a shift in inference discipline—toward interaction‐ready outputs—without being made vulnerable to the common critique that interactions cannot be inferred from metabarcoding (Barroso‐Bergadà et al. 2021; Blanchet et al. 2020). Examples of how monitoring questions map onto intended tiers and design commitments are provided in Box 1, and an operational Biomonitoring 3.0 framework is summarized in Figure 2.

BOX 1 **Biomonitoring 3.0 use‐case playbook: Design backward from the decision and the evidence tier.**

Biomonitoring 3.0 becomes operational when programs specify (i) the decision question, (ii) the interaction hypothesis, and (iii) the intended evidence tier (0–5), then adopt only the sampling and assay elements needed to meet that tier.

**Use case A: Pulse disturbance (runoff/spill/heatwave)—Tier 4–5**

- Goal: Detect exposure and a time‐lagged biological response; optionally link to a process proxy.
- Design: Event‐triggered high‐frequency sampling (hours–days), explicit lag structure, paired “source–recipient” sampling, response‐capable eRNA workflow (enrichment/targeting), plus a process proxy where feasible.
- Report as: Tier 4 unless a process consequence is demonstrated (Tier 5).
- Boundary: Best suited to events with a definable exposure window; sparse sampling without temporal alignment should not be used to support Tier 4–5 claims.

**Use case B: Biosecurity/treatment verification (ballast water, disinfection)—Tier 1–3**

- Goal: Decision‐grade detection/non‐detection with reduced legacy signal.
- Design: Before/after replication, strong blanks/controls, LOD/LOQ for targeted assays; add eRNA when dead biomass/legacy DNA is expected.
- Report as: Detection confidence/readiness; avoid mechanism claims.
- Boundary: Appropriate for detection or treatment‐verification questions, but not for inferring ecological mechanism or interaction strength.

**Use case C: Host–parasite (or disease risk) surveillance—Tier 3–4**

- Goal: Confirm interaction‐explicit links and, where possible, recipient response.
- Design: Interaction‐encoded sampling (parasite‐on‐host/host‐associated), targeted detection assays; optional host‐response transcripts with strict handling controls and time alignment.
- Report as: Tier 3 for explicit edges; Tier 4 only with aligned response and plausible alternative drivers addressed.
- Boundary: Tier 4 requires a validated recipient‐response readout and adequate temporal alignment; interaction‐explicit detection alone remains Tier 3.

**Use case D: Food‐web integrity indicators—Tier 2–5 pathway**

- Goal: Track network structure change and validate it as needed.
- Design: Seasonal/annual time series; metaweb/trait constraints; periodic interaction‐explicit validation (diet/pollen); add process measurements when aiming for Tier 5.
- Report as: Tier 1–2 unless validated by explicit edges (Tier 3) and/or signal–response/process corroboration (Tier 4–5).
- Boundary: Metaweb‐ or association‐based reconstruction alone should not be interpreted as direct evidence of realized interactions.

*Note:* Tier 1–2 needs replication + covariates; Tier 3 needs interaction‐explicit channels; Tier 4 needs paired compartments + time lags + response readouts; Tier 5 adds process consequences. Programs need not implement all components continuously; higher‐intensity modules can be added selectively or periodically according to the monitoring question and available resources.

## 5 Designing Field Programs for Interaction‐Ready, Time‐Resolved Monitoring

Sampling programs intended to support biomonitoring 3.0 have benefited from being designed around when and where interactions are expected to shift, because interaction‐ready inference can be weakened when temporal aliasing and spatial mixing have been tolerated as unavoidable noise (Deiner et al. 2016; George et al. 2024; Hallam et al. 2023; Yamahara et al. 2025). In practice, the monitoring objective has first been specified as a time scale (event‐scale hours–days, seasonal weeks–months, or long‐term years) and as a spatial domain (habitat patches, river reaches, estuaries, or landscape mosaics), after which sampling frequency and replication have been matched to the expected variance structure (George et al. 2024; Yamahara et al. 2025). High temporal variability in molecular signals has been documented in running waters, and inference has therefore been strengthened when repeated sampling has been conducted rather than relying on single‐visit snapshots (George et al. 2024; Hallam et al. 2023). Common field‐program archetypes and the tiers they can realistically support are summarized in Table 2. Implementation need not be all‐or‐none. At a lower deployment intensity, programs can build on existing molecular inventories by adding explicit interaction hypotheses, replication, and relevant covariates to support Tier 0–1 outputs. Focused seasonal or event‐based sampling and targeted interaction‐explicit assays can then support stronger Tier 2–3 claims without continuous high‐frequency monitoring. More intensive elements, including paired source–recipient sampling, response‐oriented eRNA, automation, and process measurements, can be reserved for questions requiring Tier 4–5 evidence. These components can also be deployed periodically for calibration or validation rather than maintained continuously, allowing monitoring intensity to be matched to the decision question, available resources, and required evidence strength.

**TABLE 2:** Field‐program archetypes that enable interaction‐ready, time‐resolved monitoring. Practical program patterns (archetypes) that map common monitoring objectives to feasible evidence tiers, sampling structures, molecular layers (eDNA/eRNA and transcript class), and lab/sequence strategies. The archetypes are designed to be modular: Programs can combine elements (e.g., automation + paired compartments + enrichment) to move from association‐level inference toward interaction‐explicit or signal–response evidence.

Program archetype	Intended tier(s)	Sampling pattern	Matrices & molecules	Lab/seq strategy	Best‐fit outputs	Key refs (non‐exhaustive)
High‐frequency river surveillance	1–2 (sometimes 4)	Automated + manual calibration; event‐based bursts	Water eDNA + optional eRNA	Robust controls; consistent pipeline	Time‐resolved change detection; candidate drivers	George et al. (2024), Hallam et al. (2023), Yamahara et al. (2025), Deiner et al. (2016)
Paired source–recipient monitoring	2–4	Matched sampling upstream/downstream or host/environment; explicit lags	eDNA scaffold + eRNA response	Enrichment if targeting eukaryotic response	Exposure plausibility + recipient response	Hechler et al. (2023), He et al. (2025), Scriver et al. (2025)
Biosecurity/disinfection verification	1–3	Before/after treatment; replicate controls	eDNA + eRNA to reduce legacy signal	Targeted or 16S/marker panels	Reduced “ghost” detection; compliance evidence	Xue et al. (2024), Darling et al. (2021), Klymus et al. (2020)
Food‐web indicators via metaweb constraints	2–5 (depending on validation)	Seasonal surveys + curated trophic info	eDNA inventories + metaweb	Strong taxonomy curation; sensitivity checks	Food‐web indicators (connectivity, redundancy)	D'Alessandro and Mariani (2021), Marie‐Anne Le Guen et al. (2025), Boyse et al. (2025)
Interaction‐explicit trophic monitoring	3–4	Gut/diet sampling + environment	Diet DNA + optional eRNA state	Bias‐aware thresholds; controls	Trophic edges with clear meaning	Pompanon et al. (2012), Deagle et al. (2019), Novotny et al. (2023)

Because high‐frequency field collection is rarely feasible by manual sampling alone, automation and standardization have been used as enabling design elements for time‐resolved monitoring (George et al. 2024; Yamahara et al. 2025). Autonomous sampling in lotic systems has been field‐tested and has been shown to yield detection rates comparable to manual sampling while revealing short‐term variability and associations with discharge and turbidity that would have been difficult to resolve with sparse sampling (George et al. 2024). In parallel, high‐frequency eDNA metabarcoding time series have been shown to resolve rapid changes in fish‐community composition in a large river, supporting the premise that temporal resolution can be converted into ecological insight when sufficient sampling density is achieved (Hallam et al. 2023). A state‐of‐the‐art review of aquatic eDNA sampling technologies has also emphasized that autonomous platforms can expand temporal and spatial coverage and can reduce the loss of resolution that is imposed by infrequent or logistically constrained sampling (Yamahara et al. 2025).

Interaction‐ready monitoring has also often required paired spatial designs in which potential signal sources and putative recipients are sampled in matched fashion, rather than being treated as independent biodiversity inventories (George et al. 2024; Scriver et al. 2025). In remote marine systems, paired workflows have been evaluated explicitly for combined eDNA/eRNA sampling, and comparable core biodiversity has been recovered across workflows even while method‐dependent differences have been observed for lower‐abundance taxa (Scriver et al. 2025). In that study, preservation chemistry and handling choices were shown to influence amplification success and richness estimates for fish and marine vertebrates, indicating that interaction‐ready programs should be configured around the taxa and interactions of primary interest rather than around a single universal protocol (Scriver et al. 2025).

Given the lability of RNA and the speed at which signal can be altered during field handling, preservation strategy has been treated as a design variable rather than as a technical footnote (Scriver et al. 2025; Thomas et al. 2019; Wood et al. 2020). Desiccation‐based “self‐preserving” filter housings were developed to reduce cold‐chain dependence and membrane transfer steps for eDNA workflows (Thomas et al. 2019). Buffer‐based approaches provide another practical option: RNA‐stabilization reagents have been shown to preserve filter‐bound eRNA (T. S. Jo 2023a), and DNA/RNA Shield preservation followed by combined DNA/RNA extraction has been applied in field‐oriented eDNA/eRNA workflows (Scriver et al. 2025). More recently, desiccation‐based self‐preserving filters have been evaluated in remote marine environments and have been reported to preserve eRNA well enough to recover core community composition, while buffer preservation was associated with higher richness and higher PCR success for some vertebrate targets (Scriver et al. 2025). These results have supported a pragmatic design principle: when time‐resolved interpretation is required, field workflows should be selected so that preservation performance is aligned with the target taxa and expected signal abundance, and not solely with convenience (Scriver et al. 2025; Thomas et al. 2019; Wood et al. 2020).

To reduce between‐sample variability that arises from collecting separate DNA and RNA samples, co‐collection and parallel extraction have been used in several experimental and field settings (Giroux et al. 2022; Scriver et al. 2025; Thomas et al. 2019). In marine experimental systems, both eDNA and eRNA have been quantified through time to characterize release and degradation and to highlight that persistence can be context dependent, including association with particles or surfaces (Jo 2023b; Turner et al. 2014; Wood et al. 2020). In estuarine mesocosms, DNA and RNA have been co‐extracted from sediment and compared directly as metabarcoding templates, demonstrating that community inference can shift with template choice and underscoring the need to treat paired DNA/RNA sampling as a controlled comparison rather than as interchangeable assays (Giroux et al. 2022). In field‐oriented workflow evaluations, combined eDNA/eRNA sampling has been treated as viable in remote contexts, provided that preservation and extraction decisions are made explicitly and are reported transparently (Scriver et al. 2025).

Because interaction‐ready monitoring depends on the plausibility of exposure and transport, filtration and size fractionation have been used to add interpretable structure to eRNA sampling (Hiki and Jo 2025; Jo 2023b). A comprehensive sequencing analysis of fish eRNA collected across multiple size fractions has demonstrated that eRNA can be partitioned unevenly across pore‐size ranges and that fractions can differ in the balance between target‐organism RNA and microbial RNA, supporting the use of size‐aware filtration when response readouts from macroorganisms are pursued (Hiki and Jo 2025). More generally, the “state” of environmental nucleic acids has been argued to carry information relevant to localization and temporal interpretation, with particle‐size distributions and, particularly for eDNA, fragment‐length profiles being proposed as potentially informative attributes for monitoring design and inference (Brandão‐Dias et al. 2025; Jo 2023b; Turner et al. 2014). In parallel, dissolved eRNA dynamics have been investigated experimentally and through modeling, and persistence on the order of weeks has been reported under some conditions, reinforcing that time‐resolved assumptions should be calibrated empirically rather than treated as universal (Xu and Asakawa 2025). In tube experiments, dissolved fish RNA showed half‐lives on the order of weeks (∼20–43 days) and remained detectable after 2 months under some conditions (Xu and Asakawa 2025). Binding of dissolved organic matter to RNA has also been shown to suppress nuclease‐mediated degradation, providing a plausible mechanism for longer persistence in some waters (Chatterjee et al. 2023). For routine monitoring, such calibration need not require a separate decay experiment at every site. We propose that representative calibrations spanning the relevant matrix, transcript class, season, and major environmental conditions can instead be used to define expected persistence ranges and associated uncertainty. Where suitable calibration is unavailable, temporal claims should be qualified accordingly rather than assuming a fixed eRNA detection window.

For programs intended to read out responses (Tier 4 in the evidence ladder), library preparation and enrichment strategy have been expected to determine whether response signal is detectable at all (He et al. 2025; Stevens and Parsley 2023). In outdoor freshwater mesocosms, eRNA‐based metatranscriptomics enriched for eukaryotes has been shown to capture transcriptional shifts across diverse eukaryotic taxa following acute herbicide exposure, demonstrating a practical template for field‐deployable perturbation calibration and response monitoring (He et al. 2025). In parallel, applied syntheses of eRNA have emphasized that management‐relevant monitoring is likely to require deliberate selection of gene targets, assay formats, and sequencing depth, rather than reliance on untargeted sequencing alone (Stevens and Parsley 2023). Future enrichment strategies could also explore modification‐selective aptamer capture of informative RNA fractions, particularly where reproducible differences in epitranscriptomic profiles between target and background RNA can be established (Khorshid and Ahi 2026). As a result, interaction‐ready, time‐resolved field programs have been most defensibly advanced as iterative designs in which (i) temporal sampling density is increased through automation or focused campaigns, (ii) paired sampling is used to encode interaction hypotheses, and (iii) filtration, preservation, and enrichment choices are treated as hypothesis‐linked components of the monitoring design (Hallam et al. 2023; He et al. 2025; Hiki and Jo 2025; Scriver et al. 2025; Yamahara et al. 2025).

## 6 Standards, Validation, and Translation to Decisions

Routine uptake of molecular monitoring has been limited as much by variation in how results are generated, documented, and interpreted across laboratories and programs as by sequencing throughput (Goldberg et al. 2016; Klymus et al. 2024; Nicholson et al. 2020; Ruppert et al. 2019; Zinger et al. 2019). In freshwater eDNA research, substantial gaps in metadata reporting have been documented, and a standardized reporting framework has been argued to be a practical prerequisite for reproducibility and for meaningful cross‐study synthesis (Nicholson et al. 2020). Similar concerns have been raised for metabarcoding‐based biomonitoring more broadly, where ecological conclusions have been shown to depend strongly on experimental design, controls, replication structure, and bioinformatic provenance (Blanchet et al. 2020; Ficetola et al. 2016; Zinger et al. 2019). As a result, biomonitoring 3.0 has been treated as a standards‐led proposition: interaction‐ready and time‐resolved outputs have been considered unattainable if confidence cannot be conveyed transparently and compared across sites, years, and institutions (Blanchet et al. 2020; Klymus et al. 2024; Nicholson et al. 2020; Zinger et al. 2019).

### 6.1 Minimum Information and FAIR‐By‐Design Reporting

Minimum‐information guidance has recently been consolidated for environmental metabarcoding through the MIEM guidelines, which were developed to address inconsistent documentation of laboratory configuration, sample handling, controls, bioinformatic workflows, and data archiving for both eDNA and eRNA metabarcoding (Klymus et al. 2024). In parallel, eDNA data stewardship has been recognized as a bottleneck for reuse, and metadata checklists and formatting guidance have been proposed to align eDNA outputs with the FAIR principles (findable, accessible, interoperable, reusable) (Takahashi et al. 2025; Wilkinson et al. 2016). A comprehensive FAIRe metadata checklist has been proposed to map eDNA‐specific workflow descriptors onto existing standards (e.g., MIxS and Darwin Core extensions) and to support machine‐readable interoperability across repositories and monitoring networks (Takahashi et al. 2025; Wieczorek et al. 2012; Yilmaz et al. 2011). These efforts have been directly relevant to biomonitoring 3.0 because interaction‐ready inference has been more sensitive to missing context than inventory‐based reporting; spatiotemporal resolution, sampling intensity, and covariate history have been required to interpret whether a network shift reflects ecological change rather than sampling design (Nicholson et al. 2020; Takahashi et al. 2025; Zinger et al. 2019).

A distinction has been useful between (i) minimum information for reproducibility (what is needed to re‐run a workflow and audit a result) and (ii) minimum information for inference (what is needed to justify a claim tier, such as an interaction edge or a time‐local detection) (Klymus et al. 2024; Takahashi et al. 2025). MIEM has been positioned as foundational for the former, while FAIRe‐style metadata has been designed to improve the latter by making sampling effort, spatial design, and contextual covariates discoverable and reusable (Klymus et al. 2024; Takahashi et al. 2025). For biomonitoring 3.0, it has therefore been recommended that reporting be structured around three linked “audit trails”: field sampling provenance, molecular workflow provenance, and analytical provenance (including software versions, parameters, and reference database builds) (Ahi 2025; Klymus et al. 2024; Takahashi et al. 2025). A concise set of Biomonitoring 3.0 reporting add‐ons, aligned to interaction‐ready inference, is summarized in Table 3.

**TABLE 3:** Minimum reporting add‐ons for Biomonitoring 3.0 beyond basic metabarcoding metadata. Proposed reporting elements that become critical when moving from inventories to interaction‐ready inference. Items emphasize transparency about tier intent, time‐lag structure, paired‐compartment logic, molecule/transcript class, filtration fractions, preservation timing, enrichment strategy, control structure, and provenance/versioning. These add‐ons complement MIEM/FAIRe‐style reporting by making interaction claims auditable and comparable across sites, years, and laboratories.

Reporting element (3.0 add‐on)	Why it's needed for interaction‐ready inference	Minimum to report	Applies to tier(s)	Key refs (non‐exhaustive)
Tier label (0–5)	Prevents over‐interpretation	Tier + rationale	All	Schrodt et al. (2025), Barroso‐Bergadà et al. (2021)
Interaction uncertainty & design sensitivity	Distinguishes ecological change from unstable or sampling‐dependent inferred links	Sampling density & replication; edge/network uncertainty or stability; sensitivity to resampling or alternative inference methods	1–5	Barroso‐Bergadà et al. (2021), Blanchet et al. (2020), Weiss et al. (2016), Zinger et al. (2019)
Time‐lag structure	Enables direction/signal→response tests	Lag choice, cadence, replication	2–5	Runge et al. (2019), Schrodt et al. (2025)
Paired compartments	Makes exposure plausible	Source/recipient definition + spatial logic	2–4	He et al. (2025), Hechler et al. (2023)
Molecule type & transcript class	Time resolution depends on RNA class	DNA vs. RNA; rRNA vs. mRNA; targets	1–4	Yates et al. (2021), Morgado‐Gamero et al. (2025)
Filtration/size fractions	State affects localization/transport	Pore size(s), fractions, volumes	1–4	Jo (2023a, 2023b), Hiki and Jo (2025), Turner et al. (2014)
Preservation & time‐to‐stabilization	RNA is handling‐sensitive	Preservative, times, temps, deviations (audit)	1–4	Scriver et al. (2025), Thomas et al. (2019), Klymus et al. (2024)
Enrichment/library strategy	Determines detectability of response	rRNA depletion, poly(A), capture, depth	4	Hechler et al. (2023), Stevens and Parsley (2023)
Control structure (field→bioinfo)	Supports false‐positive control	Field blanks, extraction blanks, PCR neg/pos	All	MIEM: Klymus et al. (2024), Ficetola et al. (2016)
Provenance + versioning	Avoids “method drift” looking like ecology	Pipeline, parameters, DB build/version	All	Takahashi et al. (2025), Keck et al. (2023)

### 6.2 Analytical Validity for Targeted Assays: Detection Limits, Validation Levels, and qPCR Standards

When management action is tied to detection of a focal species, analytical validity has been expected to dominate the credibility of the result (Borchardt et al. 2021; Bustin et al. 2025; Klymus et al. 2020; Thalinger et al. 2021). In targeted eDNA assays, inconsistent handling of limits of detection (LOD) and quantification (LOQ) has been identified as a recurring weakness, and standardized approaches for calculating and reporting LOD/LOQ have been proposed specifically for environmental DNA applications (Klymus et al. 2020). This recommendation has been aligned with broader laboratory best practice for quantitative PCR, where the revised MIQE 2.0 guidelines have reiterated that transparent reporting of sample handling, assay design, validation, and data analysis is necessary to support repeatability and cross‐lab comparability (Bustin et al. 2025). Digital PCR is addressed separately by the updated dMIQE2020 guidelines, which specify minimum information for the design, analysis, and reporting of quantitative dPCR experiments (Whale et al. 2020). For environmental microbiology applications, an analogous minimum‐information framework has been provided by the EMMI guidelines, which have emphasized metrology, control structure, inhibition assessment, and reporting elements tailored to qPCR/dPCR studies in complex environmental matrices (Borchardt et al. 2021). Because false‐positive and false‐negative errors can be non‐trivial in eDNA workflows, statistical approaches that explicitly estimate false‐positive rates (e.g., occupancy–detection frameworks) have also been recommended as complements to laboratory controls when designs permit (Ficetola et al. 2016; Lahoz‐Monfort et al. 2016).

To bridge laboratory performance and real‐world use, a five‐level validation scale has been developed for targeted eDNA assays, and its application to hundreds of published assays has shown that validation has often been incomplete in the literature despite increasing improvement over time (Thalinger et al. 2021). This validation‐scale framing has been important for translation because it has allowed assay readiness to be communicated as a graded attribute rather than as a binary “validated/not validated” label (Thalinger et al. 2021). Practical best practice guidance for operational eDNA/eRNA workflows has also been developed in stakeholder‐facing contexts, with a peer‐reviewed synthesis emphasizing the need for quality assurance, transparent communication of uncertainty, and fit‐for‐purpose validation when methods are used by government agencies and commercial providers (De Brauwer et al. 2023; Goldberg et al. 2016).

### 6.3 Reproducibility at Scale: Inter‐Laboratory and Proficiency Testing

Cross‐laboratory comparability has been treated as a decisive step for turning molecular monitoring into infrastructure rather than research (Klymus et al. 2024; Rodriguez et al. 2025; Thalinger et al. 2021; Vasselon et al. 2025). In a recent inter‐laboratory ring test focused on marine megafauna detection, extraction performance differences have been detected among laboratories even when total DNA yields were similar, and significant laboratory‐by‐species interactions have been reported, implying that apparently minor protocol differences can propagate into detection success under realistic environmental variation (Rodriguez et al. 2025). Such findings have supported a shift from “single‐lab optimization” toward routine intercalibration exercises as part of long‐term monitoring programs, especially when outcomes are intended to be comparable across jurisdictions (Rodriguez et al. 2025).

Comparable lessons have been drawn for metabarcoding‐based status assessment. A large proficiency testing and cross‐laboratory comparison for diatom DNA metabarcoding has been conducted to support standardization for freshwater biomonitoring, with variability across steps being treated explicitly as a barrier to transferability and ecological‐status comparability if minimum requirements are not aligned (Vasselon et al. 2025). By embedding proficiency testing in a biomonitoring context, such studies have provided a template for how biomonitoring 3.0 can be made credible: interaction‐ready outputs can be pursued only if the underlying inventories and functional readouts are reproducible across laboratories and time (Klymus et al. 2024; Rodriguez et al. 2025; Vasselon et al. 2025).

### 6.4 Bioinformatic Provenance and Reference Data as Inference‐Critical Infrastructure

For biomonitoring 3.0, it has been insufficient for wet‐lab methods to be standardized if taxonomic assignment and downstream summaries remain unstable (Blanchet et al. 2020; Klymus et al. 2024; Takahashi et al. 2025; Zinger et al. 2019). Reference database limitations have been recognized as a persistent driver of unclassified reads, false assignments, and geographic bias in metabarcoding inference, and the “seven challenges” framework has synthesized database‐related failure modes that can compromise monitoring conclusions even when laboratory workflows are strong (Keck et al. 2023). These challenges have included taxonomic conflicts, incomplete geographic coverage, inconsistent curation, and version drift—issues that can be hidden when database provenance is not reported or when pipelines are treated as black boxes (Keck et al. 2023). The relevance to biomonitoring 3.0 has been direct: interaction inference and time‐resolved interpretation can be distorted by shifting taxonomic resolution or by inconsistent assignment rules across years, which can appear as ecological change if database and pipeline versions are not controlled (Keck et al. 2023; Takahashi et al. 2025).

Accordingly, MIEM has been framed around reporting the complete analytical stack—from laboratory controls to bioinformatic parameters to data archiving—so that results can be audited and reanalyzed as tools evolve (Klymus et al. 2024). FAIRe guidance has extended this logic by proposing standardized, machine‐readable metadata and formatting so that reanalysis can be conducted at scale across repositories and across monitoring programs (Takahashi et al. 2025; Wilkinson et al. 2016). For biomonitoring 3.0, it has therefore been recommended that interaction claims be accompanied by explicit statements of (i) reference database build and coverage for focal groups, and (ii) sensitivity of key outputs to plausible alternative assignment and filtering settings (Keck et al. 2023; Klymus et al. 2024; Takahashi et al. 2025).

### 6.5 Validating Interaction‐Ready Inference: Perturbations, Calibration, and Uncertainty Tiers

Interaction‐ready monitoring has been most defensibly advanced when validation has been treated as an empirical program rather than as an aspiration (Blanchet et al. 2020; Thalinger et al. 2021; Vasselon et al. 2025; Zinger et al. 2019). For network and interaction inference, the risk of spurious links has been repeatedly tied to confounding and to insufficient replication, implying that mechanistic interpretation should be accompanied by calibration steps such as controlled perturbations, replicated time series, or interaction‐explicit evidence streams (Blanchet et al. 2020; Zinger et al. 2019). In practice, validation has been made tractable by focusing on tiered claims (from association patterns to signal–response coupling) and by requiring that stronger tiers be supported by stronger design elements, such as time‐lag structure, compartment pairing, and independent measurements of plausible drivers (Blanchet et al. 2020; Thalinger et al. 2021; Zinger et al. 2019). Interaction‐based monitoring should additionally report how robust the inferred links are to the available sampling design and analytical choices. At minimum, this should include the spatial and temporal sampling density, replication, uncertainty or stability of inferred edges or network metrics, and sensitivity to resampling or alternative inference methods, where applicable. These elements help distinguish ecological change from patterns that arise because of limited sampling or method‐dependent network reconstruction (Barroso‐Bergadà et al. 2021; Blanchet et al. 2020; Weiss et al. 2016; Zinger et al. 2019).

eRNA has introduced an additional validation burden because pre‐analytical variability (capture materials, time to stabilization, storage conditions, inhibition) can reshape transcript profiles more readily than DNA inventories if handling is inconsistent (De Brauwer et al. 2023; Klymus et al. 2024). The need for explicit control structures and transparent reporting has therefore been sharpened for eRNA deployments, particularly when “response” signatures are used as monitoring readouts (Borchardt et al. 2021; Bustin et al. 2025; De Brauwer et al. 2023; Klymus et al. 2024). Rather than being treated as a barrier, this sensitivity has been positioned as a rationale for formal QA/QC tiers: a response claim has been recommended to be reported only when the sampling‐to‐sequencing chain is sufficiently characterized to rule out handling artifacts as an alternative explanation (Borchardt et al. 2021; Bustin et al. 2025; Klymus et al. 2024).

### 6.6 Communicating Results and Moving From Outputs to Decisions

Translation to decisions has been shown to fail when uncertainty is not communicated, when method readiness is implied rather than demonstrated, or when the appropriate bounds of interpretation are not stated (Stein et al. 2024; Thalinger et al. 2021). Guidance on communicating eDNA science has emphasized that confidence levels, knowledge gaps, and reliability constraints should be articulated explicitly to avoid both overstatement and unwarranted skepticism among end‐users (Stein et al. 2024). The targeted‐assay validation scale has similarly been valuable for decision contexts because it has enabled readiness to be expressed as a graded attribute aligned with intended use (exploratory screening versus routine monitoring) (Thalinger et al. 2021).

For biomonitoring 3.0, the same communication logic has been extended from assays to claims: interaction and time‐resolved statements have been recommended to be paired with an evidence tier, a description of main uncertainty sources, and a statement of what management decision is supported at that tier (Klymus et al. 2024; Stein et al. 2024; Thalinger et al. 2021). By anchoring interaction‐ready monitoring in minimum‐information standards (MIEM and FAIRe), assay and workflow validation (MIQE 2.0, dMIQE2020, EMMI, LOD/LOQ reporting, and readiness scales), and routine interlaboratory proficiency testing, biomonitoring 3.0 has been positioned as a pathway by which environmental nucleic acids can be made decision‐relevant without being made fragile to laboratory idiosyncrasy or interpretive overreach.

## 7 Conclusion: A Roadmap and Near‐Term Priorities for Biomonitoring 3.0

Biomonitoring 3.0 has been framed as a shift in the primary monitoring output—from taxa lists toward interaction‐ready, time‐resolved ecosystem interpretation—while being built on the inventory strengths established under Biomonitoring 2.0 rather than being proposed as a replacement for it (Baird and Hajibabaei 2012; Cordier et al. 2021). It is therefore an extension of the infrastructure established under Biomonitoring 2.0, but a change in monitoring objective and inference: inventories remain foundational, while dynamics‐, interaction‐, or response‐focused hypotheses, fit‐for‐purpose evidence, explicit tiering, and decision boundaries become central. The naming has been justified by a change in the monitoring object (dynamics and interaction state) rather than by a simple increase in sequencing depth or taxonomic coverage or by the adoption of eRNA alone (Baird and Hajibabaei 2012; Bohan et al. 2017; Cordier et al. 2021). eRNA has been proposed as one possible enabling layer within this framework because biological activity and response signatures can be read from environmental matrices and because RNA persistence is often, though context dependent, shorter than DNA persistence under comparable conditions, thereby supporting more time‐local inference when rapid change is targeted (Kagzi et al. 2022; Yates et al. 2021). However, eRNA is not a defining requirement, and the framework can also be implemented through DNA‐based time series, interaction‐explicit molecular channels, environmental covariates, and process measurements.

These gains come with additional resource demands. Compared with inventory‐focused workflows, more demanding Biomonitoring 3.0 applications may require denser temporal sampling, RNA‐preserving workflows, targeted or enrichment‐based assays, greater sequencing and computational effort, more advanced statistical analysis, and expertise spanning molecular ecology, bioinformatics, and ecological inference. The additional effort is most justified when the management question benefits from information on short‐lived biological responses, interaction change, or stronger attribution beyond community membership alone; where these outputs are not required, established inventory‐based approaches may remain sufficient. Biomonitoring 3.0 should therefore be implemented selectively, with monitoring intensity matched to the decision value of the additional information (Cordier et al. 2021; Papakostas et al. 2025).

Progress toward operational 3.0 has been expected to depend on a small set of practical deliverables. First, interaction claims should be made auditable by design, through routine labeling of inference tier and by wider adoption of minimum‐information reporting that allows field design, controls, and analytical provenance to be reconstructed (Cordier et al. 2021; Klymus et al. 2024). Second, time resolution should be calibrated rather than assumed, through cross‐matrix benchmarks of RNA–DNA decay contrasts and transcript‐class dependence, with uncertainty ranges reported for real sampling contexts (Kagzi et al. 2022; Morgado‐Gamero et al. 2025; Ruppert et al. 2019; Yates et al. 2021). Third, time‐resolved sampling should be made feasible through standardized, scalable collection strategies, including automated or high‐frequency approaches where event‐scale dynamics are intended to be captured (Bohan et al. 2017; George et al. 2024). Finally, validation should be treated as an empirical program in which network and interaction hypotheses are stress‐tested through replicated time series, perturbation designs, and cross‐method corroboration, so that monitoring outputs can be translated into decisions without being inflated beyond their evidentiary base (Bohan et al. 2017; Cordier et al. 2021; Papakostas et al. 2025). If these elements are assembled as shared infrastructure, biomonitoring 3.0 can be advanced as a disciplined extension of molecular biomonitoring—one in which ecosystems are monitored not only for membership but for shifting linkages and responses that often precede visible turnover.

## Author Contributions

**Leandro Lofeu:** conceptualization (equal), investigation (equal), methodology (equal), validation (equal), visualization (lead), writing – original draft (equal), writing – review and editing (equal). **Ehsan Pashay Ahi:** conceptualization (equal), investigation (equal), methodology (equal), project administration (equal), supervision (lead), validation (equal), writing – original draft (equal), writing – review and editing (equal).

## Funding

The authors have nothing to report.

## Ethics Statement

The authors have nothing to report.

## Conflicts of Interest

The authors declare no conflicts of interest.

## Data Availability

Data sharing is not applicable to this article as no datasets were generated or analyzed during the current study.

## References

[ece374311-bib-0001] Abdala‐Roberts, L. , A. Puentes , D. L. Finke , et al. 2025. “Connecting the Dots: Managing Species Interaction Networks to Mitigate the Impacts of Global Change.” eLife 14: e98899. 10.7554/ELIFE.98899. 40198102 PMC11978301

[ece374311-bib-0002] Ahi, E. P. 2025. “eRNA‐Min: Minimum Information Standard for Environmental RNA Reporting.” 10.20944/PREPRINTS202512.1765.V1.

[ece374311-bib-0003] Ahi, E. P. , A. S. Lindeza , A. Miettinen , and C. R. Primmer . 2025. “Transcriptional Responses to Changing Environments: Insights From Salmonids.” Reviews in Fish Biology and Fisheries 35: 681–706. 10.1007/S11160-025-09928-9.

[ece374311-bib-0004] Ahi, E. P. , and T. Schenekar . 2025. “The Promise of Environmental RNA Research Beyond mRNA.” Molecular Ecology 34, no. 12: e17787. 10.1111/MEC.17787. 40329897

[ece374311-bib-0005] Allesina, S. , and S. Tang . 2012. “Stability Criteria for Complex Ecosystems.” Nature 483, no. 7388: 205–208. 10.1038/nature10832. 22343894

[ece374311-bib-0006] Baird, D. J. , and M. Hajibabaei . 2012. “Biomonitoring 2.0: A New Paradigm in Ecosystem Assessment Made Possible by Next‐Generation DNA Sequencing.” Molecular Ecology 21, no. 8: 2039–2044. 10.1111/J.1365-294X.2012.05519.X. 22590728

[ece374311-bib-0007] Barroso‐Bergadà, D. , C. Pauvert , J. Vallance , et al. 2021. “Microbial Networks Inferred From Environmental DNA Data for Biomonitoring Ecosystem Change: Strengths and Pitfalls.” Molecular Ecology Resources 21, no. 3: 762–780. 10.1111/1755-0998.13302. 33245839

[ece374311-bib-0008] Blackman, R. , M. Couton , F. Keck , et al. 2024. “Environmental DNA: The Next Chapter.” Molecular Ecology 33, no. 11: e17355. 10.1111/MEC.17355. 38624076

[ece374311-bib-0009] Blanchet, F. G. , K. Cazelles , and D. Gravel . 2020. “Co‐Occurrence Is not Evidence of Ecological Interactions.” Ecology Letters 23, no. 7: 1050–1063. 10.1111/ELE.13525. 32429003

[ece374311-bib-0010] Bohan, D. A. , C. Vacher , A. Tamaddoni‐Nezhad , A. Raybould , A. J. Dumbrell , and G. Woodward . 2017. “Next‐Generation Global Biomonitoring: Large‐Scale, Automated Reconstruction of Ecological Networks.” Trends in Ecology & Evolution 32, no. 7: 477–487. 10.1016/J.TREE.2017.03.001/ASSET/223A1B83-4D2F-4F08-9AFE-87401E39AE02/MAIN.ASSETS/GR3.JPG. 28359573

[ece374311-bib-0011] Borchardt, M. A. , A. B. Boehm , M. Salit , S. K. Spencer , K. R. Wigginton , and R. T. Noble . 2021. “The Environmental Microbiology Minimum Information (EMMI) Guidelines: QPCR and dPCR Quality and Reporting for Environmental Microbiology.” Environmental Science & Technology 55, no. 15: 10210–10223. 10.1021/ACS.EST.1C01767/ASSET/IMAGES/MEDIUM/ES1C01767_0006.GIF. 34286966

[ece374311-bib-0012] Boyse, E. , K. P. Robinson , I. M. Carr , E. Valsecchi , M. Beger , and S. J. Goodman . 2025. “Inferring Species Interactions From co‐Occurrence Networks With Environmental DNA Metabarcoding Data in a Coastal Marine Food Web.” Molecular Ecology 34, no. 7: e17701. 10.1111/MEC.17701;WGROUP:STRING:PUBLICATION. 40035351 PMC11934085

[ece374311-bib-0013] Brandão‐Dias, P. F. P. , M. Shaffer , G. Guri , K. M. Parsons , R. P. Kelly , and E. A. Allan . 2025. “Differential Decay of Multiple Environmental Nucleic Acid Components.” Scientific Reports 15, no. 1: 26791. 10.1038/s41598-025-12916-5. 40702224 PMC12287283

[ece374311-bib-0014] Bustin, S. A. , J. M. Ruijter , M. J. B. van den Hoff , et al. 2025. “MIQE 2.0: Revision of the Minimum Information for Publication of Quantitative Real‐Time PCR Experiments Guidelines.” Clinical Chemistry 71, no. 6: 634–651. 10.1093/CLINCHEM/HVAF043. 40272429

[ece374311-bib-0015] Chatterjee, A. , K. Zhang , and K. M. Parker . 2023. “Binding of Dissolved Organic Matter to RNA and Protection From Nuclease‐Mediated Degradation.” Environmental Science & Technology 57, no. 42: 16086–16096. 10.1021/ACS.EST.3C05019/SUPPL_FILE/ES3C05019_SI_001.PDF. 37811805

[ece374311-bib-0016] Cordier, T. , L. Alonso‐Sáez , L. Apothéloz‐Perret‐Gentil , et al. 2021. “Ecosystems Monitoring Powered by Environmental Genomics: A Review of Current Strategies With an Implementation Roadmap.” Molecular Ecology 30, no. 13: 2937–2958. 10.1111/MEC.15472. 32416615 PMC8358956

[ece374311-bib-0017] Costea, B. A. , Y. S. Boushehri , and E. P. Ahi . 2026. “Targeted Environmental RNA Profiling Through RNase‐Guided Depletion and Enrichment.” 10.20944/PREPRINTS202605.1744.V1.

[ece374311-bib-0018] Coyte, K. Z. , J. Schluter , and K. R. Foster . 2015. “The Ecology of the Microbiome: Networks, Competition, and Stability.” Science 350, no. 6261: 663–666. 10.1126/SCIENCE.AAD2602/SUPPL_FILE/COYTE.SM.PDF. 26542567

[ece374311-bib-0019] D'Alessandro, S. , and S. Mariani . 2021. “Sifting Environmental DNA Metabarcoding Data Sets for Rapid Reconstruction of Marine Food Webs.” Fish and Fisheries 22, no. 4: 822–833. 10.1111/FAF.12553;ISSUE:ISSUE:DOI.

[ece374311-bib-0020] Darling, J. A. , C. L. Jerde , and A. J. Sepulveda . 2021. “What Do You Mean by False Positive?” Environmental DNA 3, no. 5: 879–883. 10.1002/EDN3.194. PMC8941663 35330629

[ece374311-bib-0021] De Brauwer, M. , L. J. Clarke , A. Chariton , et al. 2023. “Best Practice Guidelines for Environmental DNA Biomonitoring in Australia and New Zealand.” Environmental DNA 5, no. 3: 417–423. 10.1002/EDN3.395.

[ece374311-bib-0022] Deagle, B. E. , A. C. Thomas , J. C. McInnes , et al. 2019. “Counting With DNA in Metabarcoding Studies: How Should We Convert Sequence Reads to Dietary Data?” Molecular Ecology 28, no. 2: 391–406. 10.1111/MEC.14734. 29858539 PMC6905394

[ece374311-bib-0023] Deiner, K. , E. A. Fronhofer , E. Mächler , J. C. Walser , and F. Altermatt . 2016. “Environmental DNA Reveals That Rivers Are Conveyer Belts of Biodiversity Information.” Nature Communications 7, no. 1: 12544. 10.1038/ncomms12544. PMC5013555 27572523

[ece374311-bib-0024] Delmas, E. , M. Besson , M. H. Brice , et al. 2019. “Analysing Ecological Networks of Species Interactions.” Biological Reviews 94, no. 1: 16–36. 10.1111/BRV.12433;REQUESTEDJOURNAL:JOURNAL:1469185X;CTYPE:STRING:JOURNAL. 29923657

[ece374311-bib-0025] Djurhuus, A. , C. J. Closek , R. P. Kelly , et al. 2020. “Environmental DNA Reveals Seasonal Shifts and Potential Interactions in a Marine Community.” Nature Communications 11, no. 1: 254. 10.1038/s41467-019-14105-1. PMC6959347 31937756

[ece374311-bib-0026] Duenser, A. , and E. P. Ahi . 2026. “Development of Transcriptional Biomarkers for Ammonia Stress in Fish.” Aquaculture International 34, no. 6: 222. 10.1007/S10499-026-02618-8.

[ece374311-bib-0027] Encinas‐Viso, F. , J. Bovill , D. E. Albrecht , et al. 2023. “Pollen DNA Metabarcoding Reveals Cryptic Diversity and High Spatial Turnover in Alpine Plant–Pollinator Networks.” Molecular Ecology 32, no. 23: 6377–6393. 10.1111/MEC.16682;ISSUE:ISSUE:DOI. 36065738

[ece374311-bib-0028] Faust, K. , and J. Raes . 2012. “Microbial Interactions: From Networks to Models.” Nature Reviews Microbiology 10, no. 8: 538–550. 10.1038/nrmicro2832. 22796884

[ece374311-bib-0029] Ficetola, G. F. , P. Taberlet , and E. Coissac . 2016. “How to Limit False Positives in Environmental DNA and Metabarcoding?” Molecular Ecology Resources 16, no. 3: 604–607. 10.1111/1755-0998.12508. 27062589

[ece374311-bib-0030] George, S. D. , A. J. Sepulveda , P. R. Hutchins , et al. 2024. “Field Trials of an Autonomous eDNA Sampler in Lotic Waters.” Environmental Science & Technology 58, no. 47: 20942–20953. 10.1021/ACS.EST.4C04970/ASSET/IMAGES/LARGE/ES4C04970_0007.JPEG. 39540890 PMC11603765

[ece374311-bib-0031] Giroux, M. S. , J. R. Reichman , T. Langknecht , R. M. Burgess , and K. T. Ho . 2022. “Environmental RNA as a Tool for Marine Community Biodiversity Assessments.” Scientific Reports 12, no. 1: 1–13. 10.1038/s41598-022-22198-w. 36273070 PMC9588027

[ece374311-bib-0032] Gold, Z. , A. O. Shelton , H. R. Casendino , et al. 2023. “Signal and Noise in Metabarcoding Data.” PLoS One 18, no. 5: e0285674. 10.1371/JOURNAL.PONE.0285674. 37167310 PMC10174484

[ece374311-bib-0033] Goldberg, C. S. , C. R. Turner , K. Deiner , et al. 2016. “Critical Considerations for the Application of Environmental DNA Methods to Detect Aquatic Species.” Methods in Ecology and Evolution 7, no. 11: 1299–1307. 10.1111/2041-210X.12595.

[ece374311-bib-0034] Guri, G. , O. R. Liu , R. P. Kelly , et al. 2025. “Quantitative, Multispecies Monitoring at a Continental Scale.” bioRxiv: The Preprint Server for Biology, 2025.10.30.685180. 10.1101/2025.10.30.685180.

[ece374311-bib-0035] Haase, P. , S. U. Pauls , K. Schindehütte , and A. Sundermann . 2010. “First Audit of Macroinvertebrate Samples From an EU Water Framework Directive Monitoring Program: Human Error Greatly Lowers Precision of Assessment Results.” Journal of the North American Benthological Society 29, no. 4: 1279–1291. 10.1899/09-183.1.

[ece374311-bib-0036] Hallam, J. , E. L. Clare , J. I. Jones , and J. J. Day . 2023. “High Frequency Environmental DNA Metabarcoding Provides Rapid and Effective Monitoring of Fish Community Dynamics.” Environmental DNA 5, no. 6: 1623–1640. 10.1002/EDN3.486.

[ece374311-bib-0037] He, X. , T. Maruki , W. B. Morgado‐Gamero , et al. 2025. “Environmental RNA‐Based Metatranscriptomics as a Novel Biomonitoring Tool: A Case Study of Glyphosate‐Based Herbicide Effects on Freshwater Eukaryotic Communities.” Molecular Ecology 34, no. 22: e70164. 10.1111/MEC.70164;SUBPAGE:STRING:FULL. 41163411 PMC12617048

[ece374311-bib-0038] Hechler, R. M. , M. C. Yates , F. J. J. Chain , and M. E. Cristescu . 2023. “Environmental Transcriptomics Under Heat Stress: Can Environmental RNA Reveal Changes in Gene Expression of Aquatic Organisms?” Molecular Ecology 34: e17152. 10.1111/MEC.17152. 37792902 PMC12186723

[ece374311-bib-0039] Hiki, K. , and T. S. Jo . 2025. “Comprehensive Sequencing of Environmental RNA From Japanese Medaka at Various Size Fractions and Comparison With Skin Swab RNA.” Environmental DNA 7, no. 3: e70137. 10.1002/EDN3.70137.

[ece374311-bib-0040] Hiki, K. , T. Yamagishi , and H. Yamamoto . 2023. “Environmental RNA as a Noninvasive Tool for Assessing Toxic Effects in Fish: A Proof‐of‐Concept Study Using Japanese Medaka Exposed to Pyrene.” Environmental Science & Technology 57, no. 34: 12654–12662. 10.1021/ACS.EST.3C03737/SUPPL_FILE/ES3C03737_SI_001.XLSX. 37585234

[ece374311-bib-0041] Jo, T. , K. Tsuri , T. Hirohara , and H. Yamanaka . 2023. “Warm Temperature and Alkaline Conditions Accelerate Environmental RNA Degradation.” Environmental DNA 5, no. 5: 836–848. 10.1002/EDN3.334.

[ece374311-bib-0042] Jo, T. S. 2023a. “Methodological Considerations for Aqueous Environmental RNA Collection, Preservation, and Extraction.” Analytical Sciences 39, no. 10: 1711–1718. 10.1007/S44211-023-00382-W/METRICS. 37326949

[ece374311-bib-0043] Jo, T. S. 2023b. “Utilizing the State of Environmental DNA (eDNA) to Incorporate Time‐Scale Information Into eDNA Analysis.” Proceedings of the Royal Society B: Biological Sciences 290, no. 1999: 20230979. 10.1098/RSPB.2023.0979/79674. PMC10229230 37253423

[ece374311-bib-0044] Kagzi, K. , R. M. Hechler , G. F. Fussmann , and M. E. Cristescu . 2022. “Environmental RNA Degrades More Rapidly Than Environmental DNA Across a Broad Range of pH Conditions.” Molecular Ecology Resources 22, no. 7: 2640–2650. 10.1111/1755-0998.13655. 35643953

[ece374311-bib-0045] Keck, F. , M. Couton , and F. Altermatt . 2023. “Navigating the Seven Challenges of Taxonomic Reference Databases in Metabarcoding Analyses.” Molecular Ecology Resources 23, no. 4: 742–755. 10.1111/1755-0998.13746. 36478393

[ece374311-bib-0046] Kelly, R. P. , A. O. Shelton , and R. Gallego . 2019. “Understanding PCR Processes to Draw Meaningful Conclusions From Environmental DNA Studies.” Scientific Reports 9, no. 1: 12133. 10.1038/s41598-019-48546-x. 31431641 PMC6702206

[ece374311-bib-0047] Khorshid, M. , and E. P. Ahi . 2026. “RNA Aptamers and Epitranscriptomics: Charting Unexplored Territories in RNA Biology.” Molecular Diagnosis & Therapy 30: 383–402. 10.1007/s40291-026-00841-w. 41838348 PMC13171942

[ece374311-bib-0048] Khorshid, M. , Z. A. Khatibani , and E. P. Ahi . 2026. “Building Field‐Ready Environmental RNA Panels.” 10.20944/PREPRINTS202605.0141.V1.

[ece374311-bib-0049] Klymus, K. E. , J. D. Baker , C. L. Abbott , et al. 2024. “The MIEM Guidelines: Minimum Information for Reporting of Environmental Metabarcoding Data.” Metabarcoding and Metagenomics 8: 489–518. 10.3897/MBMG.8.128689.

[ece374311-bib-0050] Klymus, K. E. , C. M. Merkes , M. J. Allison , et al. 2020. “Reporting the Limits of Detection and Quantification for Environmental DNA Assays.” Environmental DNA 2, no. 3: 271–282. 10.1002/EDN3.29.

[ece374311-bib-0051] Kurtz, Z. D. , C. L. Müller , E. R. Miraldi , D. R. Littman , M. J. Blaser , and R. A. Bonneau . 2015. “Sparse and Compositionally Robust Inference of Microbial Ecological Networks.” PLoS Computational Biology 11, no. 5: e1004226. 10.1371/JOURNAL.PCBI.1004226. 25950956 PMC4423992

[ece374311-bib-0052] Lahoz‐Monfort, J. J. , G. Guillera‐Arroita , and R. Tingley . 2016. “Statistical Approaches to Account for False‐Positive Errors in Environmental DNA Samples.” Molecular Ecology Resources 16, no. 3: 673–685. 10.1111/1755-0998.12486. 26558345

[ece374311-bib-0053] Littlefair, J. E. , M. D. Rennie , and M. E. Cristescu . 2022. “Environmental Nucleic Acids: A Field‐Based Comparison for Monitoring Freshwater Habitats Using eDNA and eRNA.” Molecular Ecology Resources 22, no. 8: 2928–2940. 10.1111/1755-0998.13671. 35730338 PMC9796649

[ece374311-bib-0054] Liu, C. , X. Fei , I. T. Hirayama , G. Yagi , and M. Ushio . 2026. “The Potential of Methylation Signal Detection in eDNA Toward Functional Ecological Monitoring.” Communications Biology 9, no. 1: 848. 10.1038/S42003-026-10496-2. 42321336 PMC13282381

[ece374311-bib-0055] Lofeu, L. , and E. P. Ahi . 2026. “Heterokairic Genes and the Eco‐Evo‐Devo of Timing.” Evolution & Development 28, no. 2: e70036. 10.1111/EDE.70036. 41937430 PMC13051254

[ece374311-bib-0056] Macher, T. H. , J. Arle , A. J. Beermann , et al. 2024. “Is It Worth the Extra Mile? Comparing Environmental DNA and RNA Metabarcoding for Vertebrate and Invertebrate Biodiversity Surveys in a Lowland Stream.” PeerJ 12, no. 10: e18016. 10.7717/PEERJ.18016/SUPP-15. 39465159 PMC11512801

[ece374311-bib-0057] Makiola, A. , Z. G. Compson , D. J. Baird , et al. 2020. “Key Questions for Next‐Generation Biomonitoring.” Frontiers in Environmental Science 7: 476977. 10.3389/FENVS.2019.00197/FULL.

[ece374311-bib-0058] Marie‐Anne Le Guen, C. M. , A. Raoux , S. Tecchio , et al. 2025. “Environmental DNA as a Method to Reconstruct Food Webs and Assess Ecosystem Health.” Ecological Indicators 173: 113399. 10.1016/J.ECOLIND.2025.113399.

[ece374311-bib-0059] Marshall, N. T. , H. A. Vanderploeg , and S. R. Chaganti . 2021. “Environmental (e)RNA Advances the Reliability of eDNA by Predicting Its Age.” Scientific Reports 11, no. 1: 1–11. 10.1038/s41598-021-82205-4. 33531558 PMC7854713

[ece374311-bib-0060] Miyata, K. , Y. Inoue , Y. Amano , et al. 2021. “Fish Environmental RNA Enables Precise Ecological Surveys With High Positive Predictivity.” Ecological Indicators 128: 107796. 10.1016/J.ECOLIND.2021.107796.

[ece374311-bib-0061] Miyata, K. , Y. Inoue , Y. Amano , et al. 2022. “Comparative Environmental RNA and DNA Metabarcoding Analysis of River Algae and Arthropods for Ecological Surveys and Water Quality Assessment.” Scientific Reports 12, no. 1: 19828. 10.1038/s41598-022-23888-1. 36400924 PMC9674700

[ece374311-bib-0062] Miyata, K. , Y. Kusakabe , Y. Inoue , M. Yamane , and H. Honda . 2025. “Validation of Fish Environmental RNA Metabarcoding Analysis for Ecological Surveys by Additional Traditional Field Surveys in the Naka River.” Frontiers in Ecology and Evolution 13: 1540001. 10.3389/FEVO.2025.1540001/BIBTEX.

[ece374311-bib-0063] Morgado‐Gamero, W. B. , O. Tournayre , and M. E. Cristescu . 2025. “Comparative Decay Dynamics and Detectability of eDNA and eRNA in Connected and Isolated Freshwater Mesocosms Using Digital PCR.” Molecular Ecology Resources 25, no. 8: e70028. 10.1111/1755-0998.70028. 40801081 PMC12550483

[ece374311-bib-0064] Nicholson, A. , D. McIsaac , C. MacDonald , et al. 2020. “An Analysis of Metadata Reporting in Freshwater Environmental DNA Research Calls for the Development of Best Practice Guidelines.” Environmental DNA 2, no. 3: 343–349. 10.1002/EDN3.81.

[ece374311-bib-0065] Novotny, A. , B. Serandour , S. Kortsch , B. Gauzens , K. M. G. Jan , and M. Winder . 2023. “DNA Metabarcoding Highlights Cyanobacteria as the Main Source of Primary Production in a Pelagic Food Web Model.” Science Advances 9, no. 17: eadg1096. 10.1126/SCIADV.ADG1096;SUBPAGE:STRING:FULL. 37126549 PMC10132751

[ece374311-bib-0066] Oña, L. , S. K. Shreekar , and C. Kost . 2025. “Disentangling Microbial Interaction Networks.” Trends in Microbiology 33, no. 6: 619–634. 10.1016/j.tim.2025.01.013. 40044528

[ece374311-bib-0067] Papakostas, S. , T. Schenekar , and E. P. Ahi . 2025. “Translational Biodiversity Beyond Genomics: Toward Systemic Action.” 10.32942/X23644.

[ece374311-bib-0068] Parsley, M. B. , and C. S. Goldberg . 2024. “Environmental RNA Can Distinguish Life Stages in Amphibian Populations.” Molecular Ecology Resources 24, no. 4: e13857. 10.1111/1755-0998.13857. 37593778

[ece374311-bib-0069] Pashay Ahi, E. , S. Papakostas , A. Moulistanos , and T. Schenekar . 2025. “MeRIP‐Seq Detects Temperature Related Variation in Methylated Environmental RNA in Fish.” bioRxiv: the preprint server for biology, 2025.11.03.686443. 10.1101/2025.11.03.686443.

[ece374311-bib-0070] Pawlowski, J. , L. Apothéloz‐Perret‐Gentil , and F. Altermatt . 2020. “Environmental DNA: What's Behind the Term? Clarifying the Terminology and Recommendations for Its Future Use in Biomonitoring.” Molecular Ecology 29, no. 22: 4258–4264. 10.1111/MEC.15643;WGROUP:STRING:PUBLICATION. 32966665

[ece374311-bib-0071] Pawlowski, J. , A. Bonin , F. Boyer , T. Cordier , and P. Taberlet . 2021. “Environmental DNA for Biomonitoring.” Molecular Ecology 30, no. 13: 2931–2936. 10.1111/MEC.16023. 34176165 PMC8451586

[ece374311-bib-0072] Pawlowski, J. , M. Kelly‐Quinn , F. Altermatt , et al. 2018. “The Future of Biotic Indices in the Ecogenomic Era: Integrating (e)DNA Metabarcoding in Biological Assessment of Aquatic Ecosystems.” Science of the Total Environment 637–638: 1295–1310. 10.1016/J.SCITOTENV.2018.05.002. 29801222

[ece374311-bib-0073] Pompanon, F. , B. E. Deagle , W. O. C. Symondson , D. S. Brown , S. N. Jarman , and P. Taberlet . 2012. “Who Is Eating What: Diet Assessment Using Next Generation Sequencing.” Molecular Ecology 21, no. 8: 1931–1950. 10.1111/J.1365-294X.2011.05403.X. 22171763

[ece374311-bib-0074] Ramón‐Laca, A. , K. M. Nichols , K. M. Parsons , et al. 2026. “Prospects of Multipurpose Biomonitoring for Fisheries Assessment Based on Environmental Nucleic Acids.” Journal of Fish Biology 109: 693–714. 10.1111/JFB.70484. 42126097 PMC13521752

[ece374311-bib-0075] Rodriguez, L. K. , L. De Bonis , J. McKee , et al. 2025. “Inter‐Laboratory Ring Test for Environmental DNA Extraction Protocols: Implications for Marine Megafauna Detection Using Three Novel qPCR Assays.” Metabarcoding & Metagenomics 9: 1–34. 10.3897/MBMG.9.128235.

[ece374311-bib-0076] Runge, J. , S. Bathiany , E. Bollt , et al. 2019. “Inferring Causation From Time Series in Earth System Sciences.” Nature Communications 10, no. 1: 2553. 10.1038/s41467-019-10105-3. PMC6572812 31201306

[ece374311-bib-0077] Ruppert, K. M. , R. J. Kline , and M. S. Rahman . 2019. “Past, Present, and Future Perspectives of Environmental DNA (eDNA) Metabarcoding: A Systematic Review in Methods, Monitoring, and Applications of Global eDNA.” Global Ecology and Conservation 17: e00547. 10.1016/J.GECCO.2019.E00547.

[ece374311-bib-0078] Schrodt, F. , M. Beck , J. Estopinan , et al. 2025. “Advancing Causal Inference in Ecology: Pathways for Biodiversity Change Detection and Attribution.” Methods in Ecology and Evolution 16, no. 10: 2276–2304. 10.1111/2041-210X.70131;PAGE:STRING:ARTICLE/CHAPTER.

[ece374311-bib-0079] Scriver, M. , A. Zaiko , X. Pochon , et al. 2025. “Biodiversity Monitoring in Remote Marine Environments: Advancing Environmental DNA/RNA Sampling Workflows.” Marine Environmental Research 206: 107041. 10.1016/J.MARENVRES.2025.107041. 40043465

[ece374311-bib-0080] Shelton, A. O. , Z. J. Gold , A. J. Jensen , et al. 2023. “Toward Quantitative Metabarcoding.” Ecology 104, no. 2: e3906. 10.1002/ECY.3906. 36320096

[ece374311-bib-0081] Simaika, J. P. , J. Stribling , J. Lento , et al. 2024. “Towards Harmonized Standards for Freshwater Biodiversity Monitoring and Biological Assessment Using Benthic Macroinvertebrates.” Science of the Total Environment 918: 170360. 10.1016/J.SCITOTENV.2024.170360. 38311088

[ece374311-bib-0082] Stein, E. D. , C. L. Jerde , E. A. Allan , et al. 2024. “Critical Considerations for Communicating Environmental DNA Science.” Environmental DNA 6, no. 1: e472. 10.1002/EDN3.472. PMC11110536 38784600

[ece374311-bib-0083] Stevens, J. D. , and M. B. Parsley . 2023. “Environmental RNA Applications and Their Associated Gene Targets for Management and Conservation.” Environmental DNA 5, no. 2: 227–239. 10.1002/EDN3.386.

[ece374311-bib-0084] Sugihara, G. , R. May , H. Ye , et al. 2012. “Detecting Causality in Complex Ecosystems.” Science 338, no. 6106: 496–500. 10.1126/SCIENCE.1227079/SUPPL_FILE/SUGIHARA.SM.PDF. 22997134

[ece374311-bib-0085] Taberlet, P. , E. Coissac , F. Pompanon , C. Brochmann , and E. Willerslev . 2012. “Towards Next‐Generation Biodiversity Assessment Using DNA Metabarcoding.” Molecular Ecology 21, no. 8: 2045–2050. 10.1111/J.1365-294X.2012.05470.X;REQUESTEDJOURNAL:JOURNAL:1365294X;CSUBTYPE:STRING:SPECIAL;PAGE:STRING:ARTICLE/CHAPTER. 22486824

[ece374311-bib-0086] Takahashi, M. , T. G. Frøslev , J. Paupério , et al. 2025. “A Metadata Checklist and Data Formatting Guidelines to Make eDNA FAIR (Findable, Accessible, Interoperable, and Reusable).” Environmental DNA 7, no. 3: e70100. 10.1002/EDN3.70100.

[ece374311-bib-0087] Thalinger, B. , K. Deiner , L. R. Harper , et al. 2021. “A Validation Scale to Determine the Readiness of Environmental DNA Assays for Routine Species Monitoring.” Environmental DNA 3, no. 4: 823–836. 10.1002/EDN3.189.

[ece374311-bib-0088] Thébault, E. , and C. Fontaine . 2010. “Stability of Ecological Communities and the Architecture of Mutualistic and Trophic Networks.” Science 329, no. 5993: 853–856. 10.1126/SCIENCE.1188321/SUPPL_FILE/THEBAULT.SOM.PDF. 20705861

[ece374311-bib-0089] Thomas, A. C. , P. L. Nguyen , J. Howard , and C. S. Goldberg . 2019. “A Self‐Preserving, Partially Biodegradable eDNA Filter.” Methods in Ecology and Evolution 10, no. 8: 1136–1141. 10.1111/2041-210X.13212.

[ece374311-bib-0090] Turner, C. R. , M. A. Barnes , C. C. Y. Xu , S. E. Jones , C. L. Jerde , and D. M. Lodge . 2014. “Particle Size Distribution and Optimal Capture of Aqueous Macrobial eDNA.” Methods in Ecology and Evolution 5, no. 7: 676–684. 10.1111/2041-210X.12206.

[ece374311-bib-0091] Tylianakis, J. M. , R. K. Didham , J. Bascompte , and D. A. Wardle . 2008. “Global Change and Species Interactions in Terrestrial Ecosystems.” Ecology Letters 11, no. 12: 1351–1363. 10.1111/J.1461-0248.2008.01250.X. 19062363

[ece374311-bib-0092] Valiente‐Banuet, A. , M. A. Aizen , J. M. Alcántara , et al. 2015. “Beyond Species Loss: The Extinction of Ecological Interactions in a Changing World.” Functional Ecology 29, no. 3: 299–307. 10.1111/1365-2435.12356.

[ece374311-bib-0093] Vasselon, V. , S. F. Rivera , É. Ács , et al. 2025. “Proficiency Testing and Cross‐Laboratory Method Comparison to Support Standardisation of Diatom DNA Metabarcoding for Freshwater Biomonitoring.” Metabarcoding and Metagenomics 3: 35–70. 10.3897/MBMG.9.133264. PMC11980862 40213263

[ece374311-bib-0094] Weiss, S. , W. Van Treuren , C. Lozupone , et al. 2016. “Correlation Detection Strategies in Microbial Data Sets Vary Widely in Sensitivity and Precision.” ISME Journal 10, no. 7: 1669–1681. 10.1038/ISMEJ.2015.235. 26905627 PMC4918442

[ece374311-bib-0095] Whale, A. S. , W. De Spiegelaere , W. Trypsteen , et al. 2020. “The Digital MIQE Guidelines Update: Minimum Information for Publication of Quantitative Digital PCR Experiments for 2020.” Clinical Chemistry 66, no. 8: 1012–1029. 10.1093/CLINCHEM/HVAA125. 32746458

[ece374311-bib-0096] Wieczorek, J. , D. Bloom , R. Guralnick , et al. 2012. “Darwin Core: An Evolving Community‐Developed Biodiversity Data Standard.” PLoS One 7, no. 1: e29715. 10.1371/JOURNAL.PONE.0029715. 22238640 PMC3253084

[ece374311-bib-0097] Wilkinson, M. D. , M. Dumontier , I. J. Aalbersberg , et al. 2016. “The FAIR Guiding Principles for Scientific Data Management and Stewardship.” Scientific Data 3, no. 1: 1–9. 10.1038/sdata.2016.18. PMC4792175 26978244

[ece374311-bib-0098] Wood, S. A. , L. Biessy , J. L. Latchford , et al. 2020. “Release and Degradation of Environmental DNA and RNA in a Marine System.” Science of the Total Environment 704: 135314. 10.1016/J.SCITOTENV.2019.135314. 31780169

[ece374311-bib-0099] Xu, Z. , and S. Asakawa . 2025. “Release and Degradation of Dissolved Environmental RNAs From Zebrafish Cells.” RNA Biology 22, no. 1: 1–12. 10.1080/15476286.2025.2486281. PMC12026185 40167163

[ece374311-bib-0100] Xue, Z. , W. Tian , Y. Han , et al. 2024. “Environmental RNA Metabarcoding for Ballast Water Microbial Diversity: Minimizing False Positives.” Science of the Total Environment 955: 176902. 10.1016/J.SCITOTENV.2024.176902. 39401587

[ece374311-bib-0101] Yamahara, K. M. , E. A. Allan , J. Robidart , et al. 2025. “A State‐Of‐The‐Art Review of Aquatic eDNA Sampling Technologies and Instrumentation: Advancements, Challenges, and Future Prospects.” Environmental DNA 7, no. 4: e70170. 10.1002/EDN3.70170.

[ece374311-bib-0102] Yates, M. C. , A. M. Derry , and M. E. Cristescu . 2021. “Environmental RNA: A Revolution in Ecological Resolution?” Trends in Ecology & Evolution 36, no. 7: 601–609. 10.1016/j.tree.2021.03.001. 33757695

[ece374311-bib-0103] Yilmaz, P. , R. Kottmann , D. Field , et al. 2011. “Minimum Information About a Marker Gene Sequence (MIMARKS) and Minimum Information About Any (x) Sequence (MIxS) Specifications.” Nature Biotechnology 29, no. 5: 415–420. 10.1038/nbt.1823. PMC3367316 21552244

[ece374311-bib-0104] Zhang, Y. , Y. Qiu , K. Liu , et al. 2024. “Evaluating eDNA and eRNA Metabarcoding for Aquatic Biodiversity Assessment: From Bacteria to Vertebrates.” Environmental Science and Ecotechnology 21: 100441. 10.1016/J.ESE.2024.100441. 39027464 PMC11254946

[ece374311-bib-0105] Zinger, L. , A. Bonin , I. G. Alsos , et al. 2019. “DNA Metabarcoding—Need for Robust Experimental Designs to Draw Sound Ecological Conclusions.” Molecular Ecology 28, no. 8: 1857–1862. 10.1111/MEC.15060. 31033079

[ece374311-bib-0106] Zurell, D. , L. J. Pollock , and W. Thuiller . 2018. “Do Joint Species Distribution Models Reliably Detect Interspecific Interactions From Co‐Occurrence Data in Homogenous Environments?” Ecography 41, no. 11: 1812–1819. 10.1111/ECOG.03315;WGROUP:STRING:PUBLICATION.

